# Implantable micro-scale LED device guided photodynamic therapy to potentiate antitumor immunity with mild visible light

**DOI:** 10.1186/s40824-022-00305-2

**Published:** 2022-10-18

**Authors:** Jiwoong Choi, Il Seong Lee, Ju Seung Lee, Sangmin Jeon, Wan Su Yun, Suah Yang, Yujeong Moon, Jinseong Kim, Jeongrae Kim, Seunghwan Choy, Chanho Jeong, Man Kyu Shim, Tae-il Kim, Kwangmeyung Kim

**Affiliations:** 1 Medicinal Materials Research Center, Biomedical Research Division, Korea Institute of Science and Technology (KIST), Hwarangno 14-gil 5, Seongbuk-gu, 02792 Seoul, Republic of Korea; 2 KU-KIST Graduate School of Converging Science and Technology, Korea University, 02841 Seoul, Republic of Korea; 3 School of Chemical Engineering, Sungkyunkwan University (SKKU), 16419 Suwon, Republic of Korea; 4 Department of Bioengineering, Korea University, 02841 Seoul, Republic of Korea; 5 Division of Digital Clinical Research, Korea Institute of Oriental Medicine (KIOM), 1672 Yuseongdae-ro, Yuseong-gu, Daejeon, Republic of Korea; 6 Department of Biomedical Engineering, Sungkyunkwan University (SKKU), 16419 Suwon, Republic of Korea; 7 Biomedical Institute for Convergence at SKKU (BICS), Sungkyunkwan University (SKKU), 16419 Suwon, Republic of Korea; 8 College of Pharmacy, Graduate School of Pharmaceutical Sciences, Ewha Womans University, 03760 Seoul, Republic of Korea

**Keywords:** Implantable photonic device, cancer immunotherapy, Photodynamic therapy, Immune checkpoint blockade, Cell death and immune response

## Abstract

**Background:**

Photodynamic therapy (PDT) is a promising strategy to promote antitumor immunity by inducing immunogenic cell death (ICD) in tumor cells. However, practical PDT uses an intense visible light owing to the shallow penetration depth of the light, resulting in immunosuppression at the tumor tissues.

**Methods:**

Herein, we propose an implantable micro-scale light-emitting diode device (micro-LED) guided PDT that enables the on-demand light activation of photosensitizers deep in the body to potentiate antitumor immunity with mild visible light.

**Results:**

The micro-LED is prepared by stacking one to four micro-scale LEDs (100 μm) on a needle-shape photonic device, which can be directly implanted into the core part of the tumor tissue. The photonic device with four LEDs efficiently elicits sufficient light output powers without thermal degradation and promotes reactive oxygen species (ROS) from a photosensitizer (verteporfin; VPF). After the intravenous injection of VPF in colon tumor-bearing mice, the tumor tissues are irradiated with optimal light intensity using an implanted micro-LED. While tumor tissues under intense visible light causes immunosuppression by severe inflammatory responses and regulatory T cell activation, mild visible light elicits potent ICD in tumor cells, which promotes dendritic cell (DC) maturation and T cell activation. The enhanced therapeutic efficacy and antitumor immunity by micro-LED guided PDT with mild visible light are assessed in colon tumor models. Finally, micro-LED guided PDT in combination with immune checkpoint blockade leads to 100% complete tumor regression and also establishes systemic immunological memory to prevent the recurrence of tumors.

**Conclusion:**

Collectively, this study demonstrates that micro-LED guided PDT with mild visible light is a promising strategy for cancer immunotherapy.

**Supplementary Information:**

The online version contains supplementary material available at 10.1186/s40824-022-00305-2.

## Introduction

Photodynamic therapy (PDT), a promising alternative tumor treatment option, can promote antitumor immunity by inducing immunogenic cell death (ICD) in tumor cells 1–3. The tumor cells undergoing ICD induced by PDT express several damage-associated molecular patterns (DAMPs), such as calreticulin (CRT) surface exposure; the extracellular release of high mobility group box 1 (HMGB1), adenosine triphosphate (ATP) and heat shock proteins (HSPs); and the secretion of cytokines (IFN-γ and TNF-α) 4–7. However, the practical PDT uses an intense visible light owing to the shallow penetration depth of the light into biological tissues, leading to organ dysfunction through extensive tissue damage and inflammatory responses 8. Notably, severe inflammatory responses in the tumor tissues by PDT with excessively high intensity are harmful to promote antitumor immunity because of the release of immunosuppressive cytokines from tumor cells and activation of regulatory T cells, resulting in reduced immunotherapy efficiency 9,10. In contrast, too low-intensity PDT may be insufficient to induce a therapeutic efficacy because visible light cannot penetrate the surface of tumor tissues by more than 5–10 mm 11. Therefore, an approach that can deliver visible light to deeper tumors with optimal intensity and period to induce a potent ICD and minimize side effects is crucial for PDT and its application in cancer immunotherapy.

To improve the efficiency and safety of PDT, an implantable photonic approach has emerged, which employs devices stacking organic light emitting diode (OLED), small-sized LED or wireless LED systems 12–15. The miniaturized dimensions of the devices have allowed direct implantation into the core part of the tumor tissues to efficiently control and deliver the optimal intensity of visible light deep inside the tumors 8. Therefore, these implantable photonic devices integrate distinct advantages compared to practical PDT: (i) they are implantable in the tumor sites anywhere including organs deep within the body; (ii) allow multiwavelength light emission at a lower intensity optimized for photosensitizer activation; and (iii) provide controlled light emission for proper and precise light delivery at the optimal intensity 11. Consequentially, with versatile and generalizable LED device guided PDT, it is possible to deliver the optimal intensity of the visible light in a programmable and repeatable manner, resulting in the efficient treatment of refractory tumors. However, LED device guided PDT for effective cancer immunotherapy, which potentiates the antitumor immunity to achieve complete tumor regression and prevent tumor recurrence, has not yet been adequately studied.

**Scheme 1:**
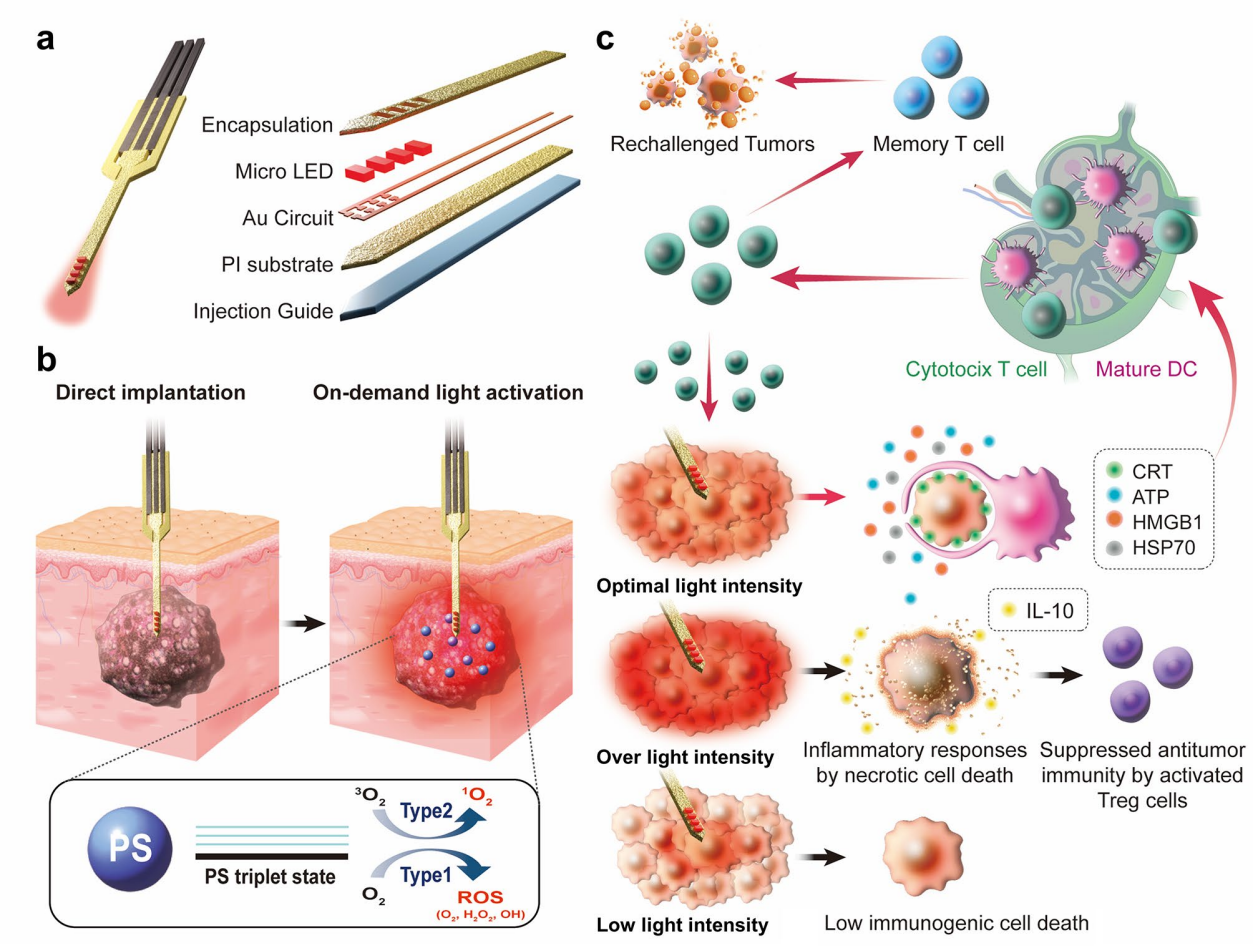
**Implantable micro-scale LED device (micro-LED) guided photodynamic therapy (PDT) to potentiate antitumor immunity with mild visible light. (a)** The micro-LED is prepared by stacking micro-scale LEDs fabricated on a polyimide (PI) substrate and transferred onto an injection guide, which was designed as a needle-like structure with polysiloxane acrylate. **(b)** The micro-LED is directly implanted into the core part of the tumor tissues, followed by irradiation with visible light. **(c)** The mechanism of micro-LED guided PDT in the tumor tissues depending on the optimal, less or over light intensity. Under the optimal light intensity, the tumor cells release DAMPs through PDT-mediated ICD, thereby promoting DC maturation and with T cell activation to inhibit the progression of primary and recurrence tumors by antitumor immunity. When over light intensity is irradiated to tumor tissues, severe inflammatory responses are induced by necrotic cell death, which releases the immunosuppressive cytokine IL-10 and activates regulatory T cells, resulting in immunosuppression. In the case of tumor tissues irradiated with too low light intensity, a potent antitumor immunity is not induced owing to the insufficient ICD in tumor cells

Herein, we propose an implantable micro-scale LED device (micro-LED) guided PDT that enables the on-demand light activation of photosensitizers deep in the body to potentiate antitumor immunity with mild visible light. The implantable photonic devices stacking a number of micro-scale LED, which allow direct implantation to the target site by needle-like structure, are prepared to deliver therapeutic doses of light in the tumor tissues inaccessible by direct illumination of outer light source. Based on its needle-like structure, these devices can be directly implanted in the inner core part of the tumor tissues, thereby allowing light activation of photosensitizers in the whole tumor tissues. In addition, the device is designed as stacking a number of LEDs with parallel array instead of a single large size one to minimize the heat generation during micro-LED guided PDT 16,17. The micro-LED is prepared by stacking one to four micro-scale LEDs (100 μm) fabricated on a 5-µm thick polyimide (PI) substrate and transferred onto an injection guide, which was designed as a needle-like structure with 100-µm thick polysiloxane acrylate (PSA; Scheme 1a**)**. Following the intravenous administration of a photosensitizer (verteporfin; VPF), the optimal intensity and period of light exposure by micro-LED implanted in the tumor tissues are investigated to promote antitumor immunity by inducing potent ICD in tumor cells **(**Scheme 1b**)**. The VPF has long been clinically used for PDT, which causes photochemical damage to the mitochondria by promoting reactive oxygen species (ROS) upon visible light irradiation with wavelength of a 690 nm 18. Scheme 1c shows the different cancer cell death mechanism using micro-LED guided PDT depending on optimal, less or over light intensity. When the optimal light intensity is irradiated to tumor tissues, the tumor cells release DAMPs and tumor-associated antigens (TAAs) through PDT-mediated ICD, thereby promoting DC maturation. The activated DCs interact with T cells in the lymph nodes to initiate antitumor immunity and then T cells attack the tumor cells, resulting in the efficient treatment of primary tumors and prevention of their recurrence by the establishment of a systemic immunological memory. In contrast, severe inflammatory responses are induced by necrotic cell death because extreme light stimulation and stress are applied to the tumor cells when over light intensity is used to irradiate tumor tissues. As a result, tumor cells release the immunosuppressive cytokine IL-10 and regulatory T cells are activated in the tumor microenvironment, which suppress the antitumor immunity. In the case of tumor tissues irradiated with too low light intensity, a potent antitumor immunity is not induced owing to the insufficient ICD in tumor cells. Hence, it is very important to properly and precisely control the light intensity of PDT for effective cancer immunotherapy. From this point of view, a micro-LED that can be precisely implanted into tumors to effectively deliver light with delicately controlled intensity could be suitable. In this study, we demonstrate that micro-LED guided PDT with the on-demand delivery of mild visible light deep into tumors in thick biological tissue provides a promising strategy for cancer immunotherapy.

## Results

### Preparation of implantable micro-scale LED device (micro-LED) for PDT

**Fig. 1:**
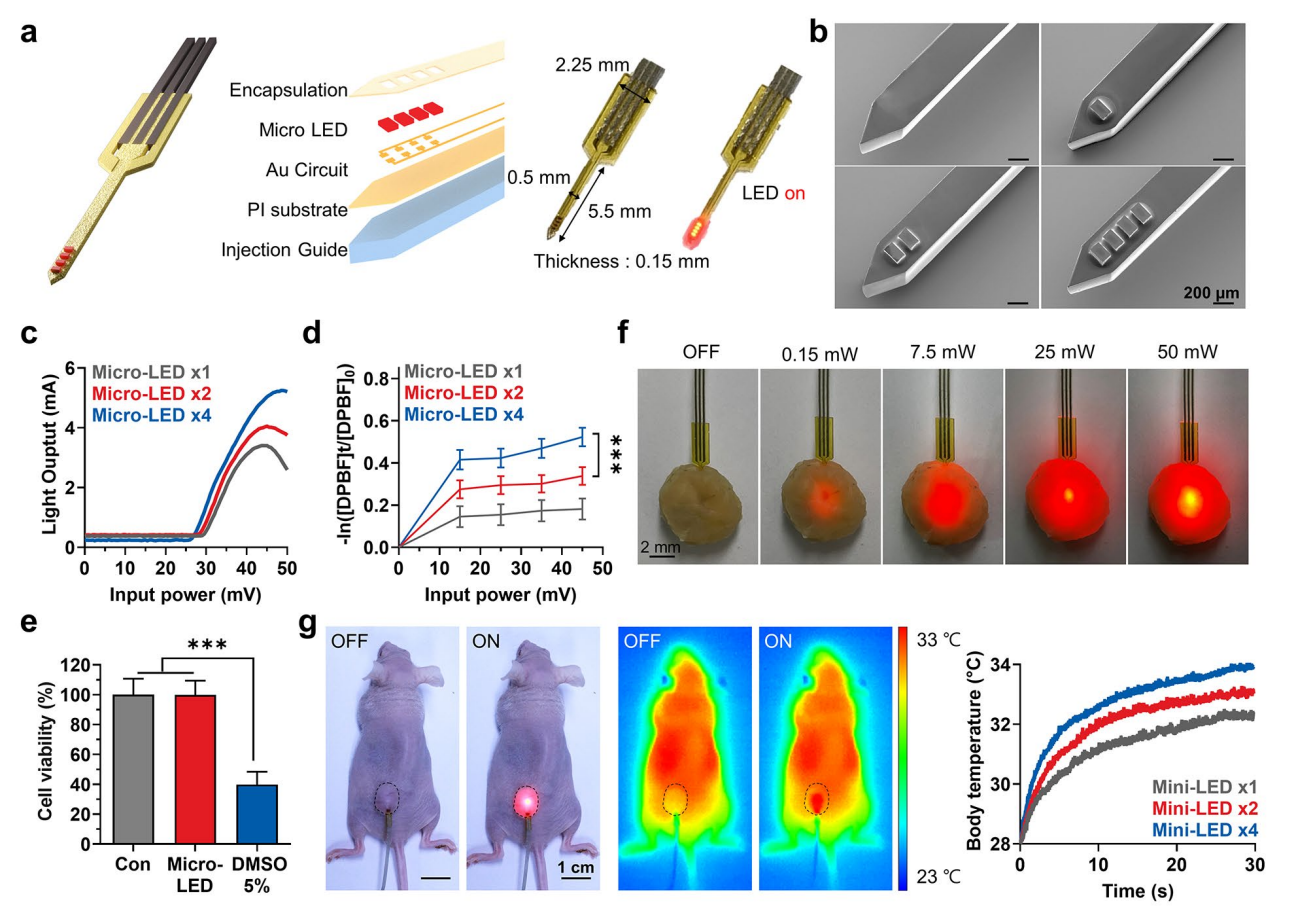
**Preparation of implantable micro-scale LED device (micro-LED) for PDT. (a)** Optical images and layout of the micro-LED. **(b)** SEM images of the micro-LED with needle-like structure and multiple LEDs. **(c)** Light output power of the micro-LED by the number of LEDs and applied voltage. **(d)** ROS generation from VPF after visible light irradiation by micro-LED with the number of LEDs and applied voltage. **(e)** The viability of L929 fibroblast cells after ISO 10993-5 cytotoxicity test of the micro-LED. **(f)** Optical images of the injection process of the micro-LED into extracted tumors. **(g)** Temperature change in thigh tumor tissue around the implanted micro-LED with 50 mW of power applied. The right panel indicates infrared thermal camera images of the mice implanted with the micro-LED. Significance was determined by Tukey − Kramer *posthoc* test

To precisely deliver the therapeutic dose of light intensity into the biological tissue, the implantable micro-scale LED device was fabricated as a needle-like structure stacking a number of LEDs (100 μm). The vertical structure and optical image of the implantable micro-scale LED device (micro-LED) are shown in Fig. 1a. It was prepared such that the assembled LED devices were fabricated on a 5-µm thick polyimide (PI) substrate and transferred onto an injection guide, which was designed as a needle-like structure with 100-µm thick PSA material **(Figure S1)**. The micro-LEDs assembled on metal (Au) patterns were interconnected by super-thin anisotropic conductive adhesive (ACA), and completely encapsulated with transparent epoxy polymer to prevent electrical interference with cells and biofluids. As can be seen from the scanning electron microscope (SEM) images, multiple micro-scale LEDs were parallelly connected in the devices **(**Fig. 1b**)**. Connecting more LEDs is not necessary because the LEDs integrated on a narrow and high aspect ratio needle area generate accumulated heat which is a critical issue in the system, and excessively strong light can induce an adverse effect on the antitumor immunity. Next, we assessed the light output powers along with the number of LEDs and applied voltage **(**Fig. 1c**)**. As the number of LEDs was doubled, the light intensity even with the same input power was almost 1.5 times stronger. Additionally, the efficiency drooping due to thermal degradation by excessive input power was much more significant when less LEDs were used and the point of drooping start when the device performance decreases was also delayed. The ROS generation from the photosensitizer VPF, irradiated by micro-LED was assessed by a bleaching test with *p*-nitroso-N,N′-dimethylaniline (RNO; Fig. 1d**)**. Under visible light irradiation for 15 min using micro-LEDs, the ROS generation from VPF was significantly upregulated in an input power-dependent manner. Importantly, micro-LED stacking four LEDs caused higher ROS generation than that with two or one LEDs at the same applied voltage. The biocompatibility of the micro-LEDs was evaluated in L929 fibroblast cells *via* the ISO 10993-5 test, wherein significant cytotoxicity was not observed because it is well-encapsulated and composed of a biocompatible material **(**Fig. 1e**)**. The optical images showing the injection process into extracted tumor tissue using the PSA injection guide demonstrated that the micro-LEDs have sufficient mechanical properties for direct implantation into the organs or tumors of living animals for the delivery of therapeutic doses of visible light **(**Fig. 1f**)**. Next, the heat generation by micro-LED devices was assessed because a high temperature can negatively influence on inducing a potent antitumor immune response by resulting inflammatory responses due to necrotic cell death in the tumor tissues 9,10. First, we assessed the heat generation by micro-LEDs under operation power of 50 mW, wherein the only 6 ^o^C was increased by the device **(Figure S2a).** In addition, the temperature change was measured when the micro-LED devices were inserted at a depth of 3 mm from the surface of a thigh tumor and operated with 50 mW input power **(**Fig. 1 g**)**. Even with applied direct current to the four parallel LEDs, the temperature in the tumor tissues rose to 34 °C. Since this is much lower than the maximum temperature of general heating-based tumor therapies that can be safely used, it can be expected that there is no damage to surrounding tissues due to the heat of the micro-LEDs. As a result, when the tumor tissues were exposed to 50 mW input power of micro-LEDs, the tumor growth was not significantly different compared to non-treated mice for 15 days **(Figure S2b)**. In addition, tumor tissues stained with H&E or TUNEL on day 15 after treatment also showed no significant structural abnormalities and apoptotic region **(Figure S2c and S2d)**. These results clearly demonstrate that there are no significant damages in the tumor tissues by heat generation of micro-LEDs. Altogether, these results confirm that the implantable micro-scale LED devices are successfully prepared and enables the on-demand light activation of photosensitizers deep into the biological tissues. Importantly, a photonic device with four LEDs efficiently elicits sufficient light output power without thermal degradation and promotes ROS from photosensitizers, and is thus suitable for delivering therapeutic dose of light intensity into the tumor tissue without adverse effects to the surrounding normal tissues.

***In vitro*characterization of micro-LED guided PDT to promote antitumor immunity**.

**Fig. 2:**
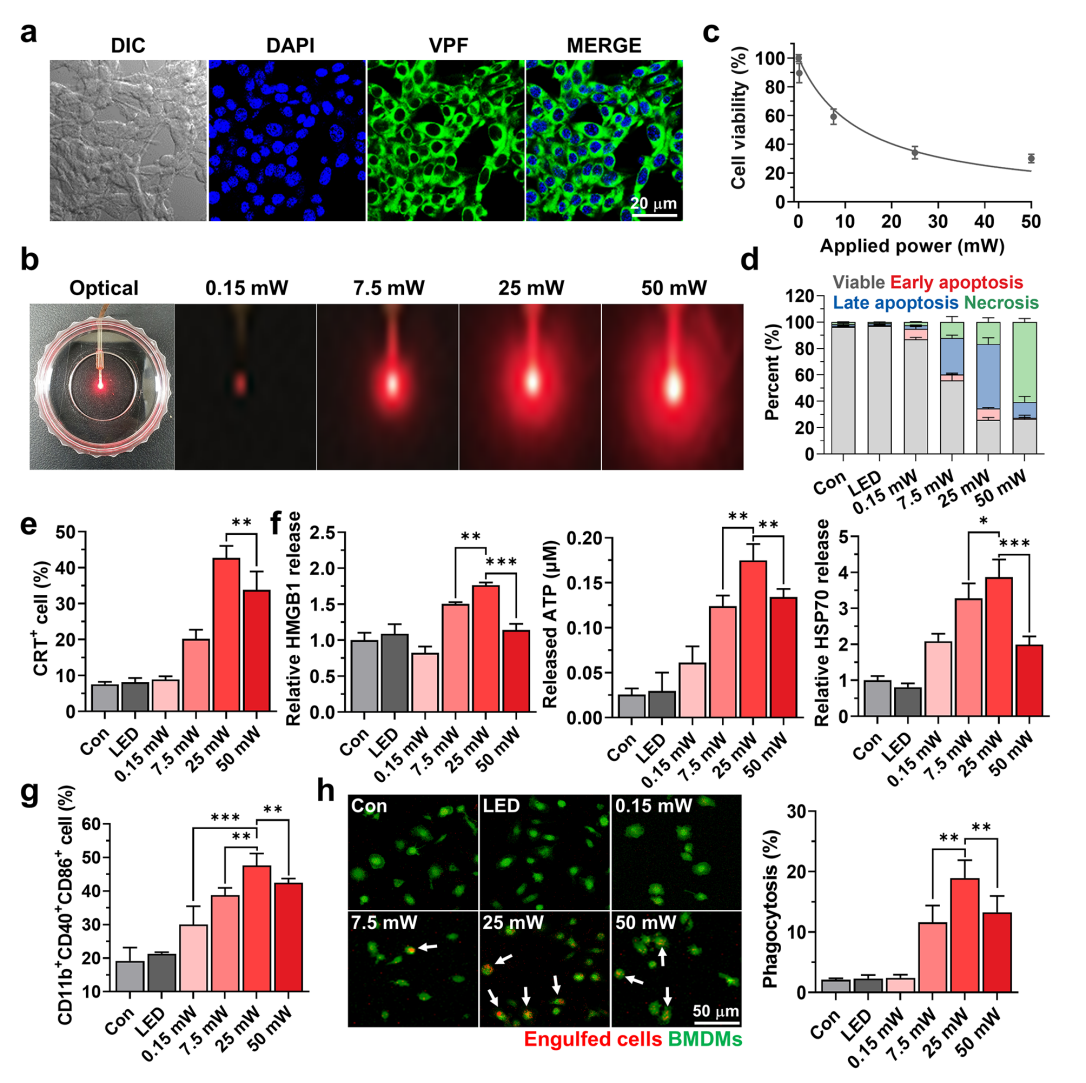
***In vitro*characterization of micro-LED guided PDT to promote antitumor immunity. (a)** Cellular uptake of VPF in the CT26 cells after 3 h of incubation at 37^o^C. **(b)** Optical images of CT26 cells irradiated with visible light by micro-LED attached to the bottom of the cell culture dishes. **(c)** The viability of the CT26 cells irradiated with different light intensities by micro-LED after 3 h of VPF treatment. **(d)** Annexin V/PI analysis of CT26 cells irradiated with different light intensities by micro-LED after 3 h of VPF treatment. **(e)** CRT expression of the CT26 cells irradiated with different light intensities by micro-LED after 3 h of VPF treatment. **(f)** Extracellular release of HMGB1, ATP and HSP70 from CT26 cells irradiated with different light intensities by micro-LED after 3 h of VPF treatment. **(g)** The percentage of mature DCs (CD11c^+^CD40^+^CD86^+^) in BMDCs after co-culture with culture medium containing CT26 cells irradiated with different light intensities by micro-LED after 3 h of VPF treatment. **(h)** CMFDA-labelled BMDMs were co-cultured with pHrodo-labelled CT26 cells irradiated with different light intensities by micro-LED after 3 h of VPF treatment. Significance was determined by Tukey − Kramer *posthoc* test

The optimal light intensity and irradiation timing that can induce potent ICD using a micro-LEDs with four LEDs were investigated in CT26 colon cancer cells. When the CT26 cells were incubated with VPF (1 µM), the fluorescence of VPF (green color) in the cytosol gradually increased until 3 h post-incubation, then significantly decreased from 6 h of incubation **(**Fig. 2a and S3**)**. Thus, the CT26 cells were exposed to different light intensities for 15 min after 3 h of VPF treatment by attaching micro-LEDs to the bottom of the cell culture dishes **(**Fig. 2b**)**. Significant cell death was not observed when the CT26 cells were exposed to micro-LEDs with 0.15 mW of power, while cell viability gradually decreased at over 7.5 mW in a light intensity-dependent manner, showing efficient property to induce cytotoxicity by promoting ROS from photosensitizers **(**Fig. 2c and S4**)**. Hence, we further performed Annexin V/PI staining to assess tumor cell death patterns after exposure to different light intensities from the micro-LED with four LEDs. The proportion of cell death was nearly similar in CT26 cells after light exposure at powers of 25 and 50 mW *via* the micro-LED, but their patterns were clearly different **(**Fig. 2d and S5**)**. When the CT26 cells were exposed to the micro-LED at a light intensity of 25 mW for 15 min, high quantities of early (8.53 ± 0.89%)/late apoptosis (49 ± 4.73%) and low necrosis (16.63 ± 3.27%) were observed. In contrast, a significantly high necrosis (60.77 ± 2.63%) and low early (0.82 ± 0.4%)/late apoptosis (11.92 ± 2.35%) were induced in the CT26 cells exposed to a light intensity of 50 mW. The unique cell death patterns in CT26 cells observed when exposed to the micro-LED with an optimal intensity of 25 mW can result in a potent ICD compared to that observed with 50 mW. This is because early apoptotic cells express CRT and phosphatidylserine on the membranes to expose “eat-me” signals, which promote the phagocytosis of DCs to upregulate the engulfment machinery 19–23. In addition, several soluble DAMPs, including HMGB1, ATP and HSP70, are released from tumor cells in a state of late apoptosis to send “find-me” signals, leading to DC maturation for the cross-presentation of tumor-associated antigens to T cells 9,24,25. However, necrosis of tumor cells is usually considered to be immunologically harmful since they expose the CD47 on the surface that induce negative engulfment “do not eat-me” signals 9,10. Therefore, annexin V/PI staining results indicate that a micro LED light intensity of 25 mW is highly favorable to elicit a potent ICD.

Next, the levels of ICD in the CT26 cells exposed to the micro-LED with different light intensities were examined by evaluating the amount of DAMPs, such as CRT surface expression and the extracellular release of HMGB1, ATP and HSP70. The VPF (1 µM) was incubated with CT26 cells for 3 h, and the cells were irradiated with light intensities ranging from 0.15 mW to 50 mW for 15 min using the micro-LED. The CRT expression on the membranes was gradually increased in the CT26 cells as the micro LED-induced light intensity was enhanced until 25 mW **(**Fig. 2e and S6**)**. Quantitatively, the quantity of CRT expression on the cell surface was increased 4.1-4.32-fold at 25 mW micro-LED-treated CT26 cells compared to that in the naive cells. However, the CRT expression in the CT26 cells decreased when the cells were exposed to the micro-LED at an intensity of 50 mW compared to that at 25 mW, which is attributable to undesirable cell death patterns of high necrosis after irradiation with over light intensity. In addition, CT26 cells treated with 25 mW of micro-LED released 1.61-1.72-fold, 1.31-1.4-fold and 2.01-2.23-fold greater HMGB1, ATP and HSP70 compared to those treated with 50 mW of micro-LED **(**Fig. 2f and S7**)**. These results indicate that the micro-LED with a light intensity of 25 mW is the most optimal condition that can promote high levels of DAMPs from the tumor cells by inducing potent ICD. We also performed co-culture assays to evaluate whether strong DAMP signals from tumor cells by micro-LED-mediated ICD lead to the maturation of DCs and phagocytosis by macrophages. First, the bone marrow-derived immature DCs were co-cultured for 24 h with culture medium containing DAMPs released from CT26 cells treated with micro-LED at different light intensities (0.15–50 mW) after incubation with VPF for 3 h. Importantly, a higher quantity of mature DCs (CD11c^+^CD40^+^CD86^+^) was observed in the 25 mW micro-LED group than other groups **(**Fig. 2 g and S8**)**. In particular, the effects promoting DC maturation were reduced when the light intensity of the micro-LED was increased from 25 mW up to 50 mW because of the increasing cell death patterns of necrosis. Similar results were also observed when the CT26 cells irradiated by micro-LED were co-cultured with bone marrow-derived macrophages (BMDMs) under the same experimental conditions. As expected, the engulfment of tumor cells (red color) by BMDMs (green color) was greatly enhanced in CT26 cells irradiated by the 25 mW micro-LED, wherein the phagocytosis (18.94 ± 2.96%) was significantly increased compared to that in other groups **(**Fig. 2 h**)**. Taken together, these results clearly demonstrate that the micro-LED with an optimal light intensity of 25 mW induce a potent ICD in tumor cells, which efficiently upregulate the DC maturation and phagocytosis of macrophages to initiate the antitumor immunity.

### Therapeutic efficacy of micro-LED guided PDT in colon tumor models

**Fig. 3:**
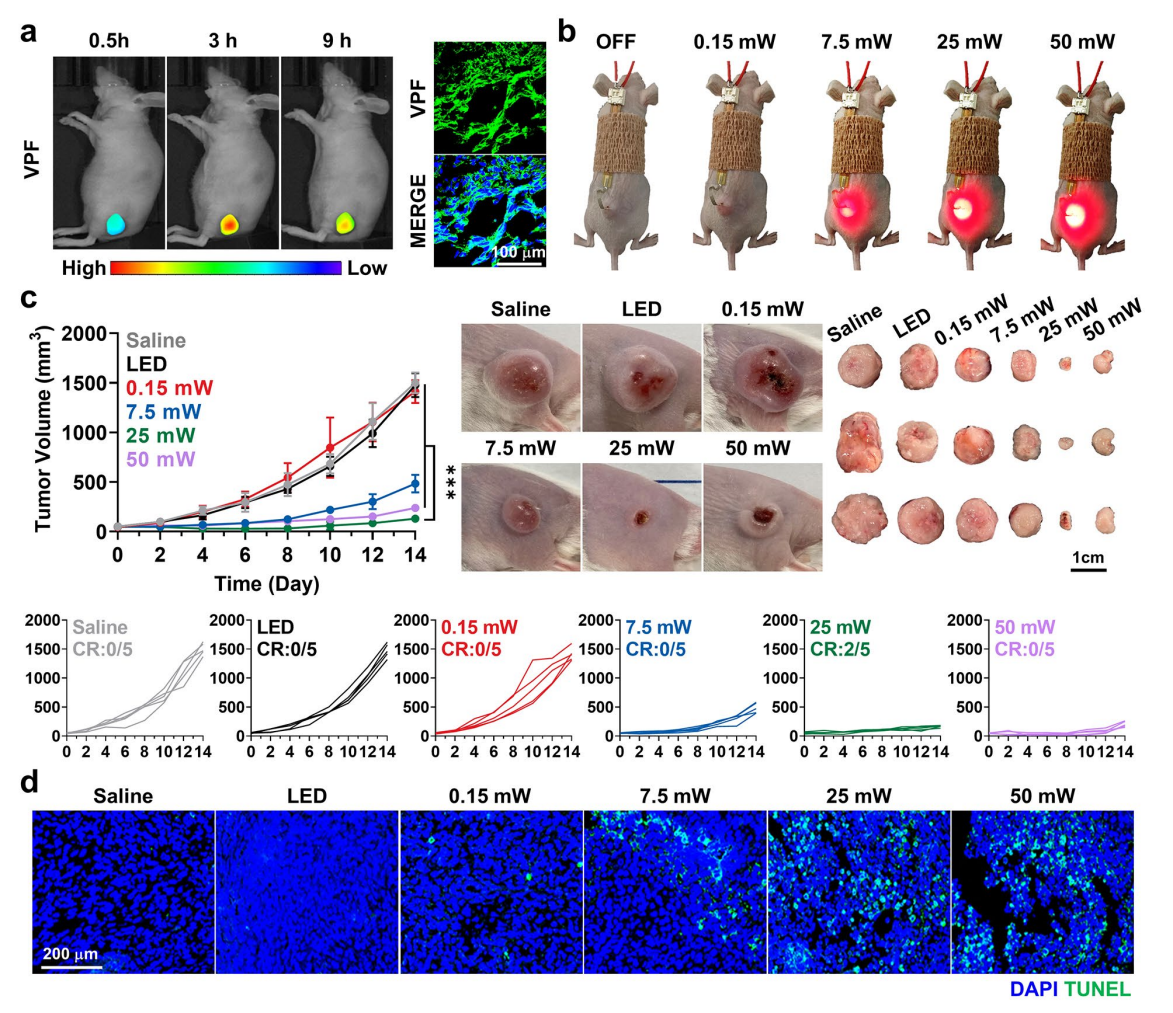
**Therapeutic efficacy of micro-LED guided PDT. (a)** Noninvasive NIRF images of murine colon tumor models treated with VPF (4 mg/kg). Right panel indicates fluorescence images of tumor tissues from mice after 3 h of VPF treatment. **(b)** Optical images of mice implanted with micro-LED for local visible light iradiation of tumor tissues. **(c)** Tumor growth of murine colon tumor models during micro-LED guided PDT at different light intensities. Right panel indicates optical images showing tumor volumes and collected tumor tissues of mice on day 14 after treatment. **(d)** Tumor tissues stained with TUNEL on day 14 after treatment. Significance was determined by Tukey − Kramer *posthoc* test

With the optimal light intensity to potentiate antitumor immunity in vitro, the therapeutic efficacy of micro-LED guided PDT was assessed in murine colon tumor models, which were prepared by the subcutaneous inoculation of 1 × 10^6^ CT26 cells. First, we found the optimal irradiation timing by evaluating tumor accumulation over time after the intravenous injection of VPF (4 mg/kg). The non-invasive near-infrared fluorescence imaging of live mice revealed the high tumor accumulation of VPF after 3 h of injection, and histological analysis of tumor tissues further showed considerable fluorescence of VPF at the same time point **(**green color; Fig. 3a**)**. Therefore, VPF (4 mg/kg) was intravenously injected into CT26 tumor-bearing mice two times with a 2 days-interval when tumor volumes were approximately 60–80 mm^3^; then, tumors were locally irradiated with different light intensities for 90 min *via* micro-LED implanted directly in the biological tissues after 3 h of drug administration **(**Fig. 3b**)**. The therapeutic efficacy was assessed by photographing and measuring the volumes of tumors. As expected, the 25 mW micro-LED group (130.88 ± 38.27 mm^3^) showed significantly delayed tumor progression on day 14 compared to mice treated with 0.15 mW (1412.09 ± 114.86 mm^3^), 7.5 mW (484.68 ± 88.11 mm^3^) and 50 mW (238.63 ± 36.31 mm^3^) of micro-LED, and saline (1501.4 ± 103.53 mm^3^) or micro-LED only **(**50 mW without VPF; 1477.53 ± 120.67 mm^3^; Fig. 3c and S9**)**. In particular, a high rate of complete tumor regression (CR: 40%) was observed in mice treated with 25 mW of micro-LED compared to those in other groups. The superior therapeutic efficacy of micro-LED guided PDT was further evaluated *via* TUNEL or H&E staining of tumor tissues on day 14 after treatment, wherein the elevated apoptosis with structural abnormalities was clearly observed in the tumors of mice treated with micro-LED guided PDT at 25 mW **(**Fig. 3d and S10**)**. From these in vivo results, we demonstated that the therapeutic efficacy of micro-LED guided PDT is significantly enhanced under the optimal light intensity of 25 mW, which is attributable to the favorable cell death patterns promoting antitumor immunity.

### Antitumor immunity by micro-LED guided PDT in colon tumor models

**Fig. 4:**
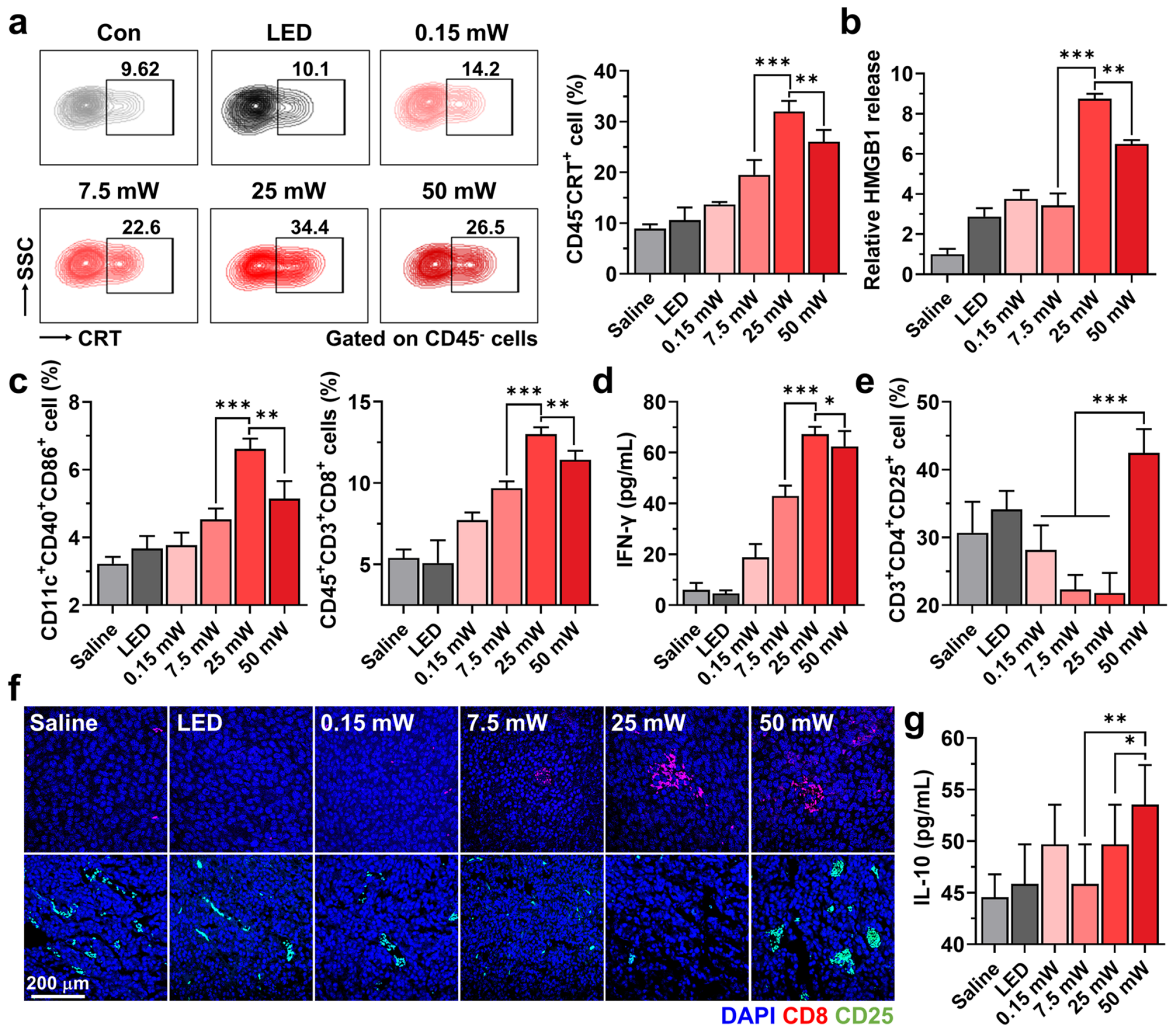
**Antitumor immunity by micro-LED guided PDT. (a)** The population of surface CRT-expressed tumor cells (CD45^-^CRT^+^) in the tumor tissues on day 14 after treatment. **(b)** Relative HMGB1 release from tumor cells to supernatants on day 14 after treatment. **(c)** Population of mature DCs (CD11c^+^CD40^+^CD86^+^) and cytotoxic T lymphocytes (CTLs; CD45^+^CD3^+^CD8^+^) in the tumor tissues on day 14 after treatment. **(d)** The amount of IFN-γ in the tumor supernatants on day 14 after treatment. **(e)** Population of regulatory T lymphocytes (Tregs; CD3^+^CD4^+^CD25^+^) in the tumor tissues on day 14 after treatment. **(f)** Tumor tissues stained with anti-CD8 or anti-CD25 antibodies on day 14 after treatment. **(g)** The amount of IL-10 in the tumor supernatants on day 14 after treatment. Significance was determined by Tukey − Kramer *posthoc* test

Next, the antitumor immunity by micro-LED guided PDT was assessed by analyzing DAMPs in tumor tissues on day 14 after treatment. The mice treated with 25 mW of micro-LED revealed significantly increased CRT-positive tumor cell (CD45^−^CRT^+^) population and HMGB1 in the tumor tissues compared to other groups **(**Fig. 4a and S11**)**. These results indicate potent ICD by micro-LED guided PDT with optimal light intensity, resulting in the recruitment of various tumor-infiltrating lymphocytes. As a result, considerably high populations of mature DCs (CD11c^+^CD40^+^CD86^+^; 6.83 ± 0.77%) and cytotoxic T lymphocytes (CTLs; CD45^+^CD3^+^CD8^+^; 12.17 ± 0.72%) were observed in the tumor tissues of mice treated with 25 mW micro-LED compared to in those treated with 0.15 mW (mature DCs: 3.77 ± 0.37% and CTLs: 7.72 ± 0.47%), 7.5 mW (mature DCs: 4.53 ± 0.32% and CTLs: 9.68 ± 0.43%) and 50 mW (mature DCs: 4.11 ± 0.87% and CTLs: 8.31 ± 0.71%) micro-LEDs, and saline (mature DCs: 5.14 ± 0.52% and CTLs: 11.43 ± 0.55%) or micro-LED only (50 mW without VPF; mature DCs: 3.67 ± 0.37% and CTLs: 5.09 ± 1.39%) groups **(**Fig. 4b and S12a**)**. Furthermore, the high activity of CTLs in the tumor tissues from mice treated with 25 mW micro-LED was evaluated by confirming an upregulated IFN-γ concentration in the tumor supernatants **(**Fig. 4c**)**. In contrast, the quantity of regulatory T lymphocytes (Tregs; CD3^+^CD4^+^CD25^+^) in the tumor tissues was significantly decreased in the 25 mW micro-LED-treated group **(**Fig. 4d and S12b**)**. The high CTLs (red color) and low Tregs (green color) in the tumors of mice treated with 25 mW of micro-LED was further confirmed *via* histological analysis **(**Fig. 4e**)**. Most importantly, the Tregs in the tumor tissue of mice treated with 50 mW micro-LED was considerably increased compared to that in all other groups. This is attributable to severe inflammatory responses due to necrotic cell death in the tumor tissues, which cause the activation of Tregs, due to over light intensity. In addition, IL-10, an immunosuppressive cytokine in the tumor microenvironment, was significantly increased in mice treated with 50 mW micro-LED than in those treated with 25 mW micro-LED, showing adverse effects by inflammatory responses owing to excessively high light intensity **(**Fig. 4f**)**. Taken together, the appropriate light intensity and irradiation timing of micro-LED guided PDT are optimized in vivo, which potentiate a potent antitumor immunity in the body by recruiting TILs to induce an immune-favorable tumor microenvironment.

### Combination of micro-LED guided PDT and immune checkpoint blockade in colon tumor models

**Fig. 5:**
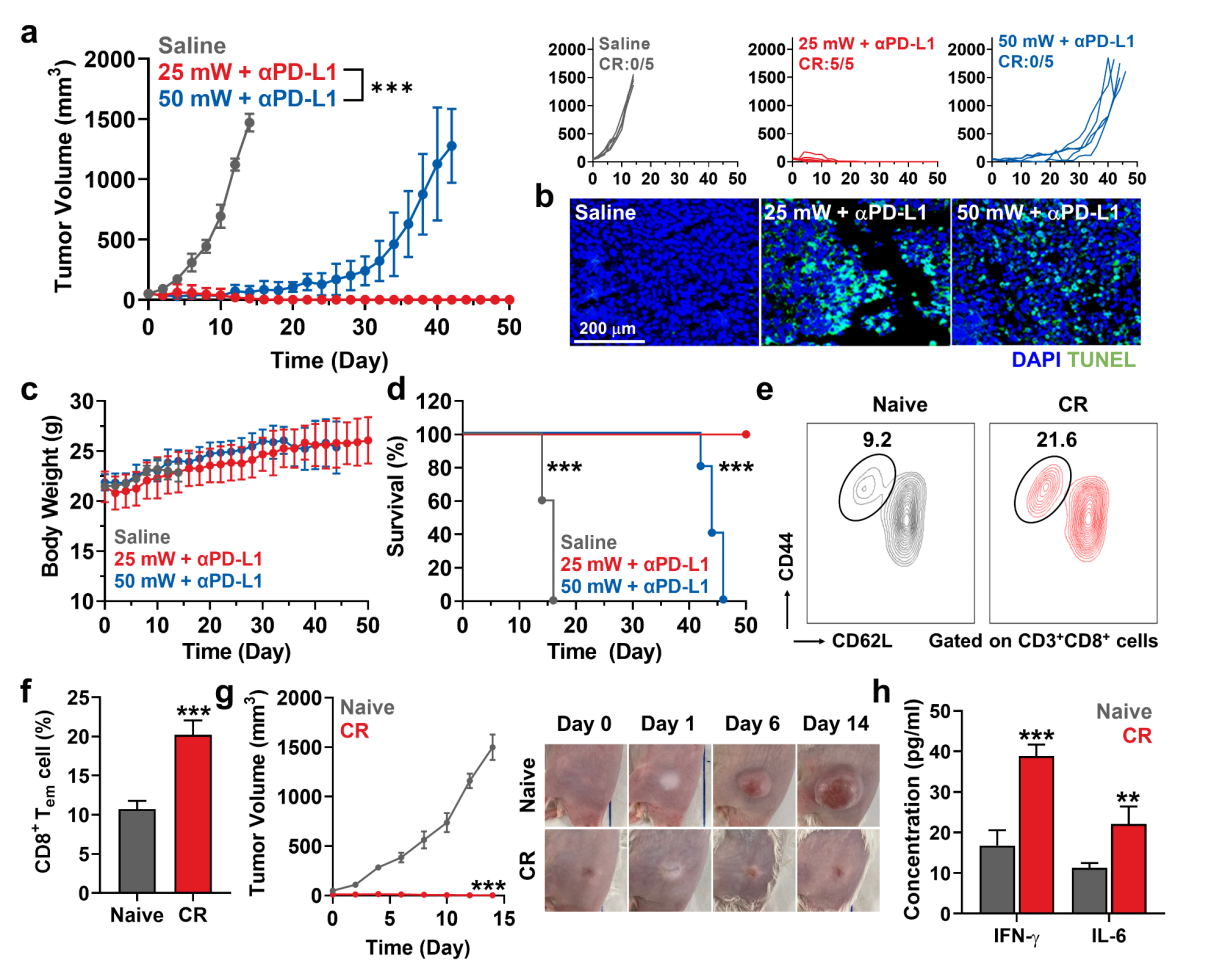
**Combination of micro-LED guided PDT and immune checkpoint blocakde. (a)** Tumor growth of murine colon tumor models during co-treatment with micro-LED guided PDT and immune checkpoint blockade. **(b)** Tumor tissues stained with TUNEL on day 14 after treatment. **(c)** Body weight change during treatment. **(d)** Mice survival during treatment. **(e, f)** The population of splenic effector/memory T cells among the CD8^+^ T cells (CD3^+^CD8^+^CD44^+^CD62L^low^) in mice that experienced complete tumor regression (CR) by treatment with 25 mW micro-LED and anti-PD-L1 antibody. **(g)** Tumor growth and optical images of CR and naive mice rechallenged with CT26 cells. **(h)** The amount of IFN-γ and IL-6 in the serum after 15 days of tumor rechallenge. Significance was determined by Student’s *t* test (f-h), log-rank test (d) or Tukey − Kramer *posthoc* test (a)

The successful optimization of micro-LED guided PDT prompted us to investigate whether its combinatorial treatment with immune checkpoint blockade resulted in a high rate of complete tumor regression. This is because PDT can induce ICD in tumor cells to potentiate antitumor immunity, but tumor cells in the process of ICD also promote the negative feedback mechanism of PD-L1 on the cell surface to evade the immune responses 26,27. Accordingly, several ICD-inducing modalities were extensively combined with the anti-PD-L1 antibody in preclinical and clinical studies, which showed a promising outcome with multiple cases in complete tumor regression 28,29. Therefore, we investigated the enhanced therapeutic efficacy and antitumor immune responses by combinatorial treatment with micro LED-guided PDT and anti-PD-L1 antibody in colon tumor models. The CT26 tumor-bearing mice were divided into three groups: saline, 25 mW micro-LED with anti-PD-L1 antibody (αPD-L1), and 50 mW micro-LED with αPD-L1. The VPF (4 mg/kg) was intravenously injected into the mice twice with a 2 days-interval, and αPD-L1 (10 mg/kg) was simultaneously i.p. injected; then, tumors were locally irradiated with 25 mW or 50 mW of light intensity by micro-LED after 3 h of VPF injection. As expected, a combination of 25 mW micro-LED and αPD-L1 significantly inhibited tumor growth compared to that in other groups **(**Fig. 5a and S13**)**. The tumor tissues stained with TUNEL further revealed elevated apoptotic cell death in mice treated with 25 mW micro-LED and αPD-L1 **(**Fig. 5b**)**. During treatments, significant body weight changes and toxicity in the major organs were not observed in all groups, wherein the structural abnormalities in the major organs were assessed after H&E staining **(**Fig. 5c and S14**)**. Importantly, treatment with 25 mW micro-LED and αPD-L1 achieved 100% complete tumor regression (CR: 5/5) for up to 50 days, while mice treated with 50 mW micro-LED and αPD-L1 were all dead within 40 days owing to continuous tumor progression **(**Fig. 5d**)**. These indicate the reduced immunotherapy efficiency due to over light internsity that causes the release of immunosuppressive cytokines from tumor cells and activates the regulatory T cells by severe inflammatory responses.

Next, the population of effector/memory T lymphocytes (T_em_; CD3^+^CD8^+^CD44^+^CD62L^low^), hallmarks of adaptive immunity, was assessed in mice that experienced complete tumor regression (CR mice) through 25 mW micro-LED and αPD-L1 to evaluate the establishment of immunological memory that prevents recurrence by previously encountered tumor cells. As a result, a significant downregulation of CD62L in the CD8 + T lymphocytes was observed in CR mice, wherein the population of splenic T_em_ cells was significantly upregulated (20.2 ± 1.85%) compared to those in naive mice **(**10.74 ± 1.04%; Fig. 5e and f**)**. In addition, CR mice were further rechallenged with CT26 tumor cells on day 50 after treatment; interestingly, the results revealed that CR mice were resistant to rechallenged tumor growth for 14 days, whereas those rapidly grew in naive mice **(**Fig. 5 g**)**. Finally, the levels of cytokines, such as IFN-γ and IL-6 in the serum were greatly increased in CR mice compared to those in naive mice after tumor rechallenge, showing strong antitumor immunity by immunological memory to prevent the recurrence of tumors **(**Fig. 5 h**)**. Based on our findings, this study demonstrated that micro-LED guided PDT with optimal light intensity and irradiation timing can induce 100% tumor complete regression by enhanced therapeutic efficacy and antitumor immunity when combined with immune checkpoint blockade. In addition, their combinatorial treatment subsequently establish a tumor-specific adaptive immunity to prevent the recurrence of tumors.

## Discussion

To overcome the fundamantal problems of shallow penetration depth of the light in photodynamic therapy (PDT), an implantable photonic approach has emerged in recent decades. The current important benefirts of PDT is that it can induce a potent antitumor immune response in body by promoting immunogenic cell death (ICD) in tumor cells 1–3. However, practical PDT was forced to use intense power of light due to the depth limitation in the biological tissues; more importantly, intense light power can lead to a immunosuppression in the tumor microenvironment owing to necrotic cell death resulting in inflammatory responses 9,10. For eliciting a potent antitumor immune responses in the tumor tissues, it is necessary to induce a early or late apoptosis that promote “find-me” signals for DC maturation to present tumor-associated antigens to T cells 9,24,25. Consequentially, new approach that is possible to efficiently induce a apoptotic death of tumor cells by delivering the optimal intensity of the visible light in a programmable and repeatable manner is urgently needed for effective PDT-mediated cancer immunotherapy.

In present study, an implantable micro-scale LED device (micro-LED) guided PDT that enables the on-demand light activation of photosensitizers deep in the body was proposed. From the optimization studies, micro-LED stacking four LEDs efficiently elicited light output without thermal degradation compared to that with one or two LEDs. Importantly, we minimized the heat generation from devices by a parallel array of several micro-LEDs instead of a single large size one. Connecting several micro-LEDs in parallel can effectively control the heat generation without current drooping 16,17. Thus, this micro-LEDs was suitable for delivering therapeutic dose of light intensity into the tumor tissue without damages to the surrounding normal tissues. Therefore, the necrotic cell death that hinder in inducing an antitumor immune response *via* inflammatory responses could be also minimized.

Using the micro-LEDs, optimal condition to elicit high early and late apoptosis in tumor cells was carefully optimized in cell culture system. When irradiation power of micro-LEDs was increased to 25 mW, the proportion of apoptosis in tumor cells was gradually increased, but low apoptosis with high necrosis in tumor cells was observed at tumor cells exposed to 50 mW power of micro-LEDs. In addition, CRT surface exposure and release of HMGB1 and ATP were significantly higher in tumor cells irradiated with 25 mW power of micro-LEDs, resulting in upregulated maturation and phagocytic activity of DCs when those tumor cells were co-cultured with DCs. Similar results were also observed in colon tumor-bearing mice. We found that the tumor growth was significantly inhibited when the tumor tissues were irradiated by micro-LEDs with irradiation power of 25 mW. This indicate that micro-LEDs can efficiently eradicate the tumor tissues with minimal power of light by efficiently delivering the therapeutic dose of the light to deep inside of the tumor tissues.

Under optimal condition of micro-LED guided PDT, colon tumor-bearing mice undergo a potent ICD in tumor tissues, which resulted in high levels of DAMPs in the tumor cells. As a result, the levels of mature DCs and cytotoxic T lymphocytes were significantly increased in tumor tissues, whereas regulatory T lymphocytes were greatly reduced compared to tumor tissues exposed to intense power of 50 mW micro-LED. In addition, the combination of immune checkpoint blockade with micro-LED guided PDT resulted in 100% complete tumor regression in the colon tumor-bearing mice by promoting a considerable antitumor immune response. Finally, the combination of immune checkpoint blockade with micro-LED guided PDT also prevented the tumor regression, wherein the mice that experienced the complete tumor regression showed delayed tumor growth from rechallenged tumors owing to established adoptive immune responses in vivo.

## Conclusion

In this study, we proposed the promising and alternative approach to potentiate antitumor immunity *via* micro-LED guided PDT. The implantable micro-scale LED device was fabricated to properly and precisely deliver the optimal therapeutic dose of light intensity. Designed as a needle-like structure, the micro-LED device has desirable mechanical properties for direct implantation into the biological tissue and thus allows the on-demand light activation of photosensitizers deep in the body with control sustainably the light exposure of constant intensity. In addition, appropriate light intensity and irradiation timing to promote potent antitumor immunity by inducing ICD in tumor cells were optimized. High early and late apoptosis of tumor cells were induced when the tumor cells were irradiated with a light intensity of 25 mW by micro-LED, whereas light intensity of more than 25 mW resulted in necrotic cell death. With favorable cell death patterns induced by the micro-LED at the optimal light intensity, colon tumor-bearing mice experienced complete tumor regression at a high rate of 40% owing to a strong antitumor immunity, which recruits cytotoxic T lymphocytes and excludes regulatory T lymphocytes in the tumor microenvironment. Finally, a combination of micro-LED guided PDT and immune checkpoint blockade achieved 100% complete regression of primary tumors through enhanced therapeutic efficacy and antitumor immunity and efficiently established an adaptive immunity that promotes systemic immunological memory, preventing tumor recurrence. This study clearly demonstrates that micro-LED guided PDT with the on-demand delivery of mild visible light deep into the tumor site provides a promising strategy for cancer immunotherapy.

## Materials and methods

**Reagents.** Verteporfin (VPF) was purchased from Frontier Scientific (Logan, UT, USA). The TUNEL assay kit was purchased from R&D systems (Minneapolis, MN, USA). The Annexin V/PI staining kit was purchased from Sigma Aldrich (Ontario, Canada). Dulbecco’s modified Eagle medium (DMEM) high glucose, RPMI 1640 medium, fetal bovine serum, penicillin and streptomycin were purchased from WELGENE Inc. (Daegu, Republic of Korea). Anti-mouse high mobility group box 1 (HMGB1), anti-mouse heat shock protein 70 (HSP70), anti-mouse calreticulin (CRT) and anti-β-actin antibodies were purchased from Abcam (Hanam, Republic of Korea). Fluorescent dye-conjugated antibodies against mouse CD45, mouse CD44, mouse CD8a, mouse CD40, mouse CD62L, mouse CD11c, mouse CD3 and mouse CD86 were purchased from BioLegend (San Diego, CA, USA). Anti-mouse PD-L1 antibody (B7-H1) was purchased from BioXCell (Lebanon, NH, USA). CT26 (mouse colon adenocarcinoma) was purchased from American Type Culture Collection (ATCC; Manassas, VA, USA). The polysiloxane acrylate was purchased from MCnet (Gyeonggi-do, Republic of Korea). The photoresist epoxy, SU-8 2, was purchased from MicroChem (Round Rock, TX, USA).

**Fabrication of implantable micro-scale LED device (micro-LED).** Two pieces of spacers composed of 100-µm thick polyethylene terephthalate (PET) were placed between a cleaned bare glass substrate and a photomask patterned in an injection guide shape. After polysiloxane acrylate (PSA) precursor was poured carefully and filled the gap by capillary force, it was pre-cured by UV flood exposure (250–400 nm wavelength with 60 mJ/cm^2^) into an injectable guide to help the implantation of the bio-injectable device. The upper photomask layer was mechanically peeled off, followed by washing out of the uncured PSA precursor with isopropanol. Additional UV irradiation (10 mJ/cm^2^ for 12 h) was applied to the PSA injectable guide to get fully cured mechanical property. After the cleaning process of a glass substrate, a 50-nm thick poly (methyl methacrylate) (PMMA, MicroChem, USA) sacrificial layer and a 5-µm thick polyimide precursor (poly-(pyromellitic dianhydride-co-4,4-oxydianiline), amic acid solution, Sigma-Aldrich Inc., USA) were sequentially coated using a spin-coater (Spin-1200D, Midas system, Korea). For thermal curing, the coated substrate was annealed in vacuum oven for 5 min at 150 °C and 2 h at 250 °C. After fully curing, metal layers (Cr/Au = 5/100 nm) of electrodes were deposited by thermal evaporation and patterned by conventional photolithography and wet-etching methods. Four micro-LEDs were assembled on anisotropic conductive adhesive-coated electrodes by a deterministic transfer printing process using a transparent polydimethylsiloxane (PDMS) elastomer. Then, 2-µm thick UV-curable, negative photoresist epoxy, SU-8 2, was used to encapsulate the LEDs. Using a plasma etcher, residual PI and PMMA was etched using a Cu etch mask and immersed in acetone to remove the PMMA sacrificial layer under the PI substrate. The needle-like etched PI layer with four micro-LEDs was mechanically separated from the glass, and transferred onto the PSA injectable guide.

**Characterization of the micro-LED.** The micro-LEDs were characterized using a digital camera (EOS 60D DLSR, Canon Inc., Tokyo, Japan), an optical microscope (DM2700M; Leica Microsystems GmbH, Wetzlar, Germany), and a field emission SEM (JSM-7600 F; JEOL Ltd., Tokyo, Japan). The thermal properties of the micro-LED were measured using an infrared camera (T420; FLIR System Inc., Wilsonville, OR, USA). The electrical properties were evaluated using a probe station (MST 5500B, MS Tech, Seoul, Korea) and precision source/measure unit (B2901A; Agilent Technologies Inc., Santa Clara, CA, USA). Optical properties of micro-LED were estimated by a light spectrometer (HR4000; Ocean Optics Inc., Dunedin, FL, USA).

**Cytotoxicity test.** The In vitro cytotoxicity test was performed using the ISO 10993-5 method to investigate the biocompatibility of the micro-LED. First, L929 fibroblast cells (Korean Cell Line Bank) were cultured in DMEM supplemented with 10% (v/v) fetal bovine serum and 1% (v/v) antibiotic-antimycotic at 37 °C in a humidified atmosphere of 5% CO_2_. At confluence, L929 cells were cultivated in a 20% (v/v) extract of the micro-LED that was infused in DMEM at 37 °C for 24 h. Then, the surviving L929 cells were evaluated by colorimetric assay using a 10% (v/v) cell counting kit-8 (Dojindo, Kumamoto, Japan) at 450 nm. The tissue culture plate and 5% (v/v) DMSO were adopted as negative and positive controls, respectively.

**Cellular uptake.** For fluorescence imaging, 1 × 10^5^ CT26 cells were seeded in 35-mm glass-bottom confocal dishes, followed by incubation with VPF (1 µM) at 37°C. After incubation, cells were washed with DPBS two times, fixed with 4% paraformaldehyde for 15 min, and stained with 4’,6-diamidino-2-phenylindole (DAPI) for 10 min. Fluorescence imaging was performed *via* a Leica TCS SP8 laser-scanning confocal microscope (Leica Microsystems GmbH) equipped with diode (405 nm), Ar (458, 488, 514 nm), and He-Ne (633 nm) lasers.

**DAMP analysis.** The CRT surface expression, and extracellular release of HMGB1, ATP and HSP70 were analyzed to assess the DAMPs from tumor cells. First, 2 × 10^5^ CT26 cells were seeded in 35-mm cell culture dishes, followed by incubation with VPF (1 µM) for 3 h at 37 °C. Then, cells were irradiated with visible light using micro-LED attached to the bottom of the dishes for 15 min. Then, supernatants were collected to analyze the HMGB1, ATP and HSP70 released from the cells. The ATP in the cell culture medium was analyzed using a commercialized ATP assay kit (Beyotime Biotechnology, Jiangsu, China). HMGB1 and HSP 70 were analyzed *via* western blot. In addition, cells were stained with FITC-conjugated CRT antibodies for 24 h at 4^o^C, followed by analysis using flow cytometer (BD FACSVerse, BD Bioscience, USA) and Leica TCS SP8 laser-scanning confocal microscope. To assess the populations of apoptosis and necrosis after drug treatment and visible light irradiation using the same protocol as described above, cells were incubated with binding buffer containing Annexin V-FITC (5 µg) and propidium iodide (PI; 10 µg) for 15 min at 37^o^C. Then, the quantities of cell apoptosis and necrosis were analyzed *via* flow cytometer (BD FACSVerse, BD Bioscience, USA).

**Co-culture assay.** To assess the maturation of DCs and phagocytosis of macrophages, 2 × 10^6^ CT26 cells were seeded in 100-pi cell culture dishes. Following 24 h of stabilization, cells were treated with VPF (1 µM) for 3 h, followed by visible light irradiation at intensities of 0–50 mW for 15 min using the micro-LED. Then, CT26 cells and the medium containing released DAMPs after each treatment were further co-cultured with BMDCs or BMDMs from BALB/c mice for 24 h. Finally, mature DCs (CD11c^+^CD40^+^CD86^+^) and tumor cell phagocytosis by BMDMs were analyzed using a flow cytometer (BD FACSVerse, BD bioscience, USA) and Leica TCS SP8 laser-scanning confocal microscope (Leica Microsystems GmbH), respectively.

**Therapeutic efficacy of micro-LED guided PDT.** Five-week-old male BALB/c nu/nu and BALB/c mice were purchased from NaraBio (Gyeonggi-do, Republic of Korea). Mice were bred under pathogen-free conditions at the Korea Institute of Science and Technology (KIST). All experiments with live animals were performed in compliance with the relevant laws and institutional guidelines of the Institutional Animal Care and Use Committee (IACUC) in KIST, and the IACUC approved the experiment (approved number 2020 − 123). Murine colon tumor models were prepared by subcutaneous inoculation of 1 × 10^6^ CT26 cells into the flanks of the mice and divided into six groups: (i) saline, (ii) LED, (iii) 0.15 mW, (iv) 7.5 mW, (v) 25 mW and (iv) 50 mW. Then, the mice were treated with VPF (4 mg/kg) twice with a 2 days-interval. When the tumor volume was 60–80 mm^3^, tumor tissues were irradiated with each intensity for 90 min using the micro-LED. In the LED group, tumor tissues were irradiated with 50 mW without VPF treatment. Tumor volumes were calculated as the largest diameter x smallest diameter^2^ × 0.53, every 2 days. For the analysis of antitumor immunity, tumor tissues were collected from mice on day 14, followed by the isolation of single cells from tissues through a Tumor Dissociation Kit (Miltenyi Biotec). After cell counting, single cells were incubated with FcBlock for 10 min to avoid non-specific antibody binding. Then, multi-parameter staining was performed for 40 min at 4^o^C to identify the following populations in tumor tissues; (i) CRT^+^ tumor cells (CD45^−^CRT^+^), (ii) cytotoxic T lymphocytes (CTLs; CD45^+^CD3^+^CD8^+^), (iii) mature DCs (CD11c^+^CD40^+^CD86^+^) and (iv) regulatory T cells (Tregs; CD3^+^CD4^+^CD25^+^).

To assess the enhanced therapeutic efficacy and antitumor immunity by combinatory micro-LED guided PDT and immune checkpoint blockade, anti-PD-L1 antibody (10 mg/kg) was simultaneously administered with VPF *via* intraperitoneal injection. On day 50 after treatment, the systemic immunological memory in CR mice that experience complete tumor regression (CR) was evaluated by tumor rechallenge with CT26 cells (1 × 10^6^). Rechallenged tumor volumes were measured every 2 days, and splenic effector/memory CD8^+^ T cells, defined as CD3^+^CD8^+^CD44^+^CD62L^low^, were analyzed by flow cytometry. Finally, IFN-γ and IL-6 in the serum were analyzed using an ELISA kit at the endpoint (day 65 after first tumor inoculation).

**Statistics.** The statistical significance between two groups was analyzed using Student‘s *t*-test. One-way analysis of variance (ANOVA) was performed for comparisons of more than two groups, and multiple comparisons were analyzed using the Tukey–Kramer post hoc test. Survival data was plotted as Kaplan–Meier curves and analyzed using the log-rank test. The statistical significance was indicated with asterisks (*p < 0.05, **p < 0.01, ***p < 0.001) in the figures.

## Electronic supplementary material

Below is the link to the electronic supplementary material.

Supplementary Material 1

Supplementary Material 2

## Data Availability

All relevant data are available within the article and its supplementary information files, or available from the corresponding authors upon reasonable request.
