# Supplementary material for: Implantable micro-scale LED device guided photodynamic therapy to potentiate antitumor immunity with mild visible light
Source: Biomater Res. 2022 Oct 18;26:56. doi: 10.1186/s40824-022-00305-2 (PMC9580183; doi:10.1186/s40824-022-00305-2)
Supplement: Supplementary file 1 — Supplementary Material 1 [file 40824_2022_305_MOESM1_ESM.docx]

**Supplementary Information for**

Implantable micro-scale LED device guided photodynamic therapy

to potentiate antitumor immunity with mild visible light

Jiwoong Choi^1,2,#^, Il Seong Lee^1,2,#^, Ju Seung Lee^3,#^, Sangmin Jeon^1^, Wan Su Yun^1,2^, Suah Yang^1,2^, Yujeong Moon^1,4^, Jinseong Kim^1,2^, Jeongrae Kim^1,2^, Seunghwan Choy^5^, Chanho Jeong^6^, Man Kyu Shim^1,*^, Tae-il Kim^3,7,*^, Kwangmeyung Kim^1,2,8,*^

^1^Center for Theragnosis, Biomedical Research Institute, Korea Institute of Science and Technology (KIST), Hwarangno 14-gil 5, Seongbuk-gu, Seoul 02792, Republic of Korea.

^2^KU-KIST Graduate School of Converging Science and Technology, Korea University, Seoul, 02841, Republic of Korea.

^3^School of Chemical Engineering, Sungkyunkwan University (SKKU), Suwon 16419, Republic of Korea.

^4^Department of Bioengineering, Korea University, Seoul, 02841, Republic of Korea.

^5^Division of Digital Clinical Research, Korea Institute of Oriental Medicine (KIOM), 1672 Yuseongdae-ro, Yuseong-gu, Daejeon, Republic of Korea.

^6^Department of Biomedical Engineering, Sungkyunkwan University (SKKU), Suwon, 16419, Republic of Korea.

^7^Biomedical Institute for Convergence at SKKU (BICS), Sungkyunkwan University (SKKU), Suwon 16419, Republic of Korea.

^8^College of Pharmacy, Graduate School of Pharmaceutical Sciences, Ewha Womans University, Seoul 03760, Republic of Korea.

^#^These authors contributed equally to this work.

*Corresponding authors: **K. Kim** (E-mail: kimkm@ewha.ac.kr), **T. I. Kim** (E-mail: taeilkim@skku.edu) and **M. K. Shim** (E-mail: [mks@kist.re.kr](mailto:kim@kist.re.kr)).

**
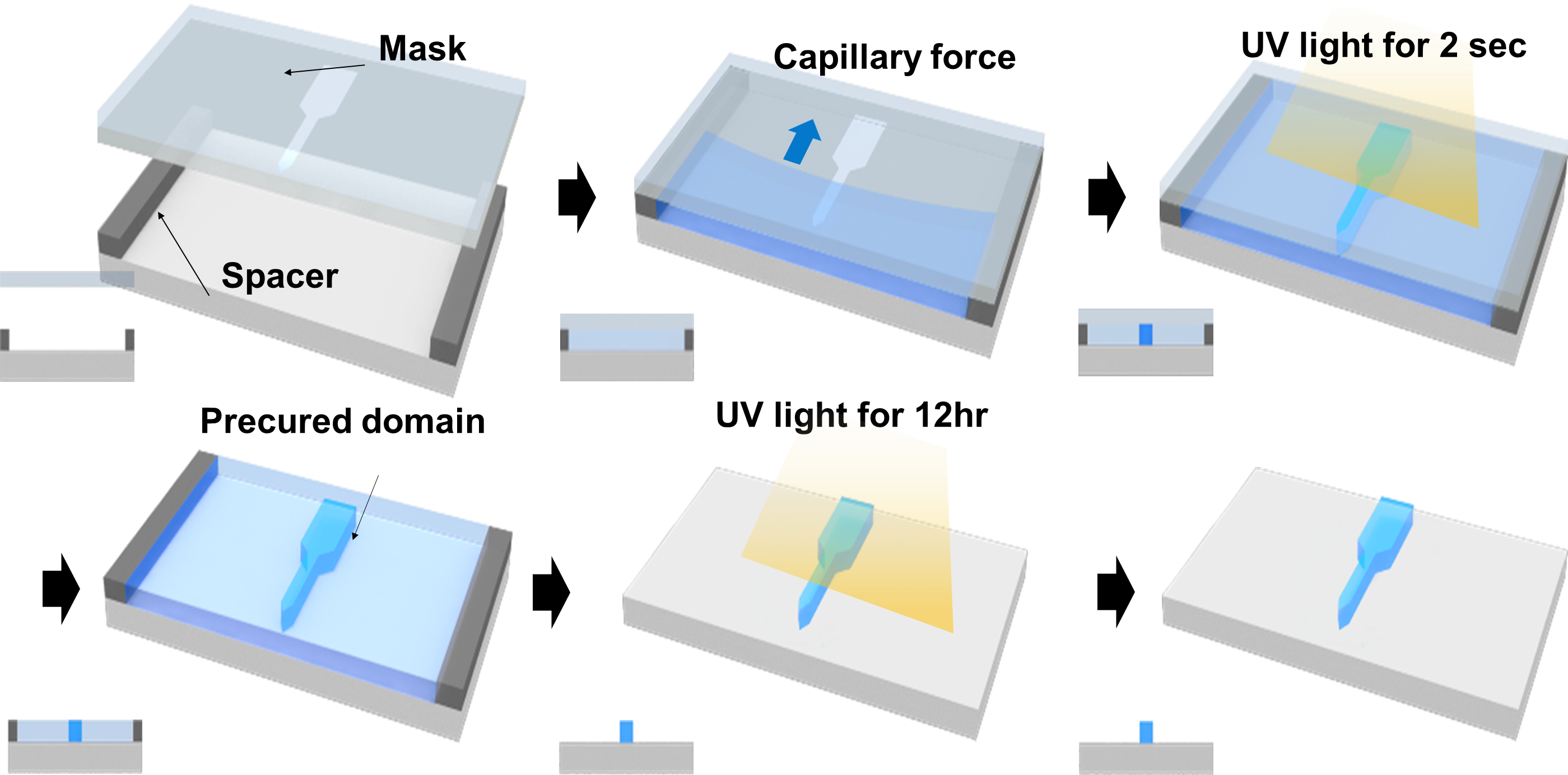
**

**Figure S1.** Schematic illustration to show the preparation protocol of the micro-LEDs.


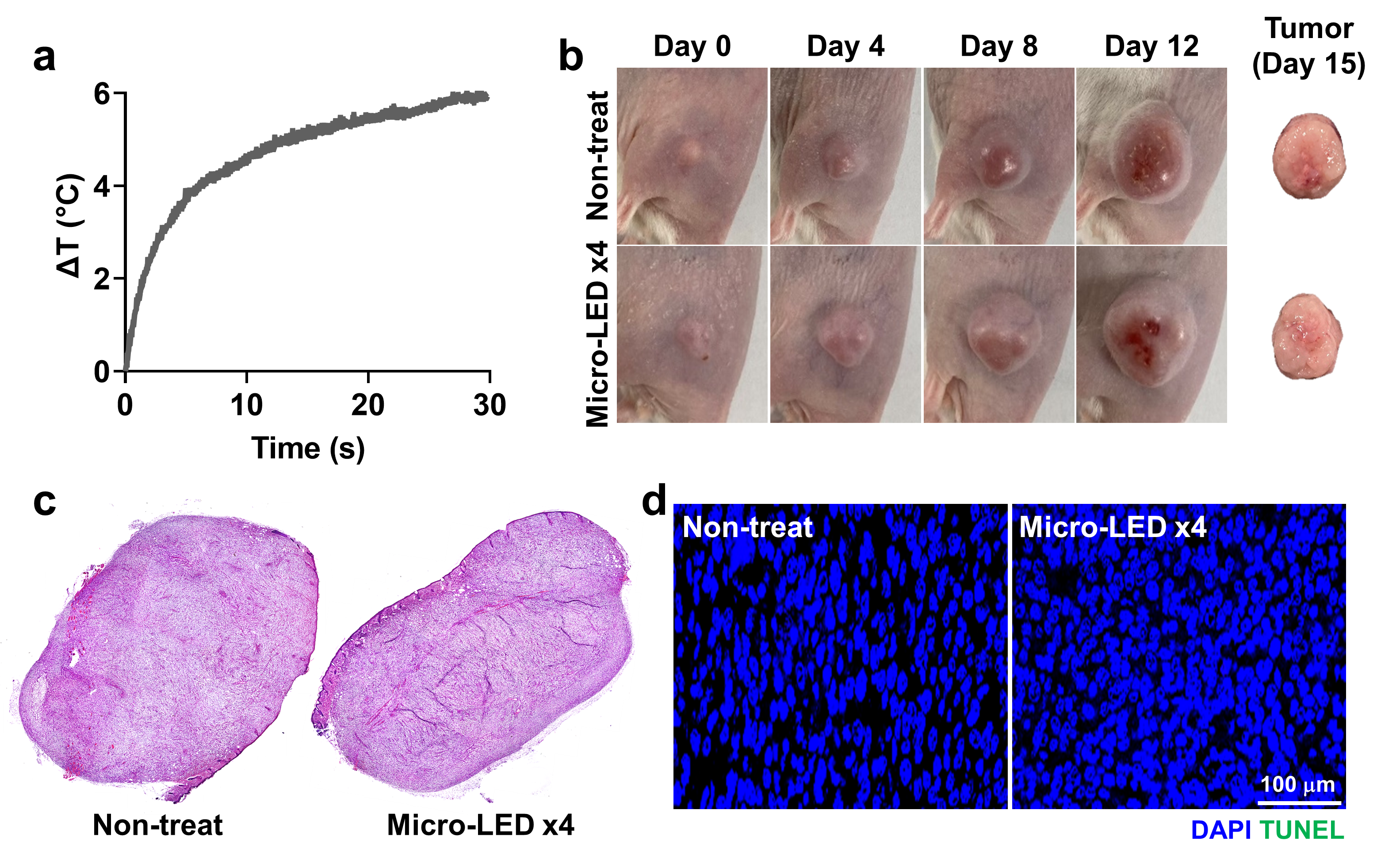


**Figure S2.** **(a)** Heat generation by micro-LEDs under input power of 50 mW. **(b)** The tumor growth of CT26 tumor-bearing mice when the tumor tissues were exposed to 50 mW input power of micro-LEDs. **(c-d)** The tumor tissues stained with **(c)** H&E or **(d)** TUNEL on day 15 after treatment.


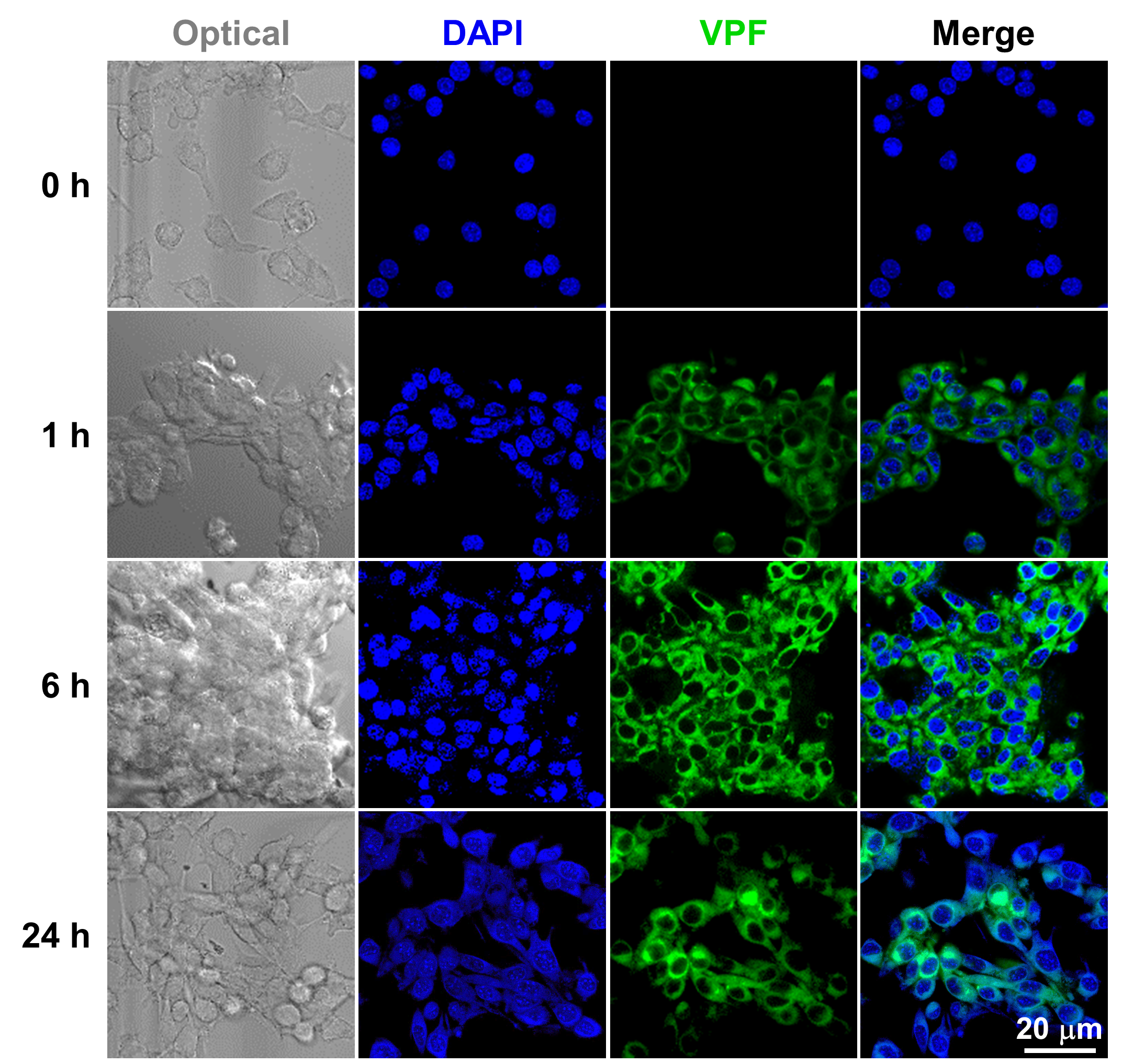


**Figure S3.** Cellular uptake of VPF in the CT26 cells after 0, 1, 6 or 24 h of incubation at 37^o^C.


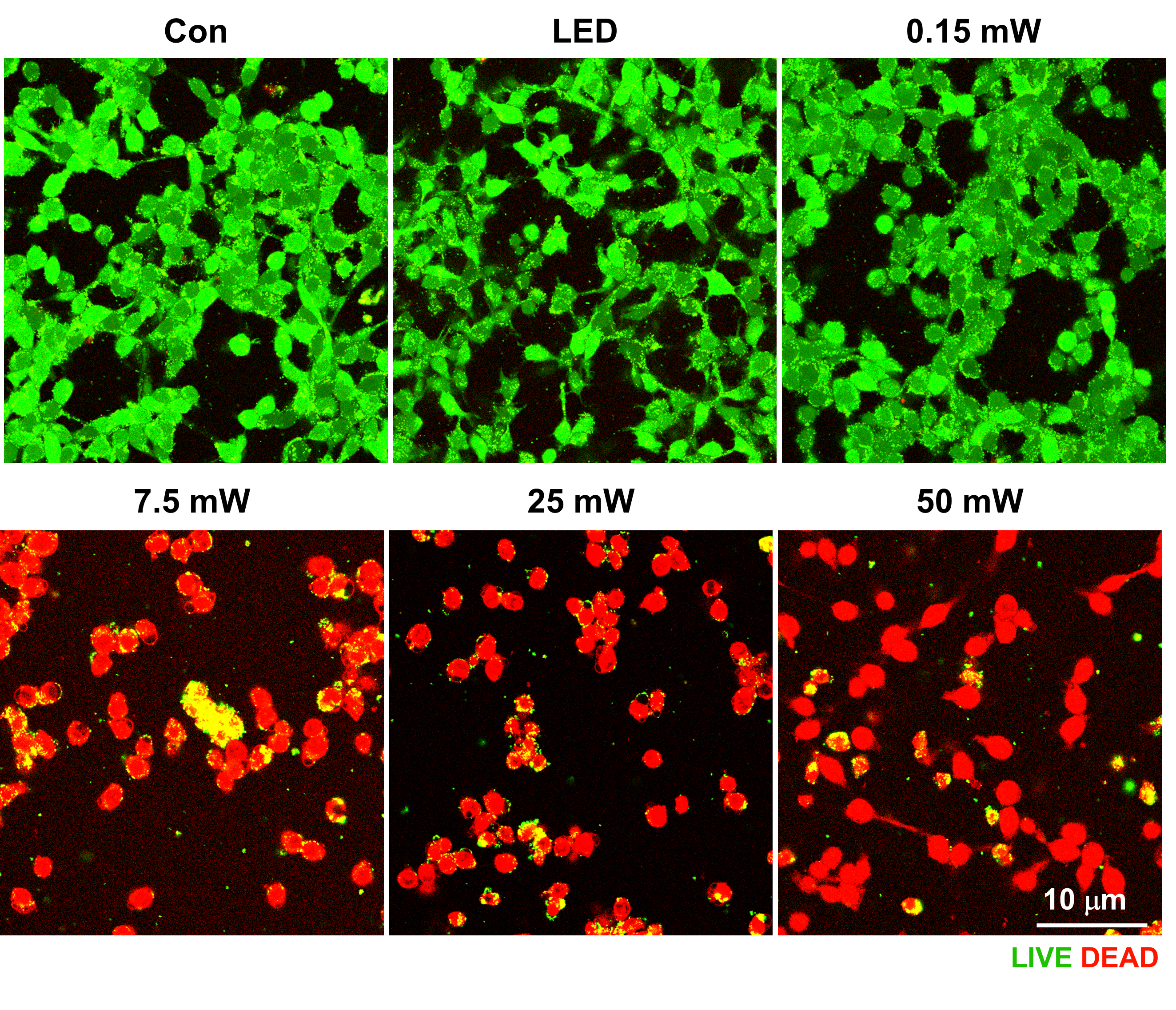


**Figure S4.** Live/Dead analysis of CT26 cells irradiated with different light intensities by micro-LED after 3 h of VPF treatment.


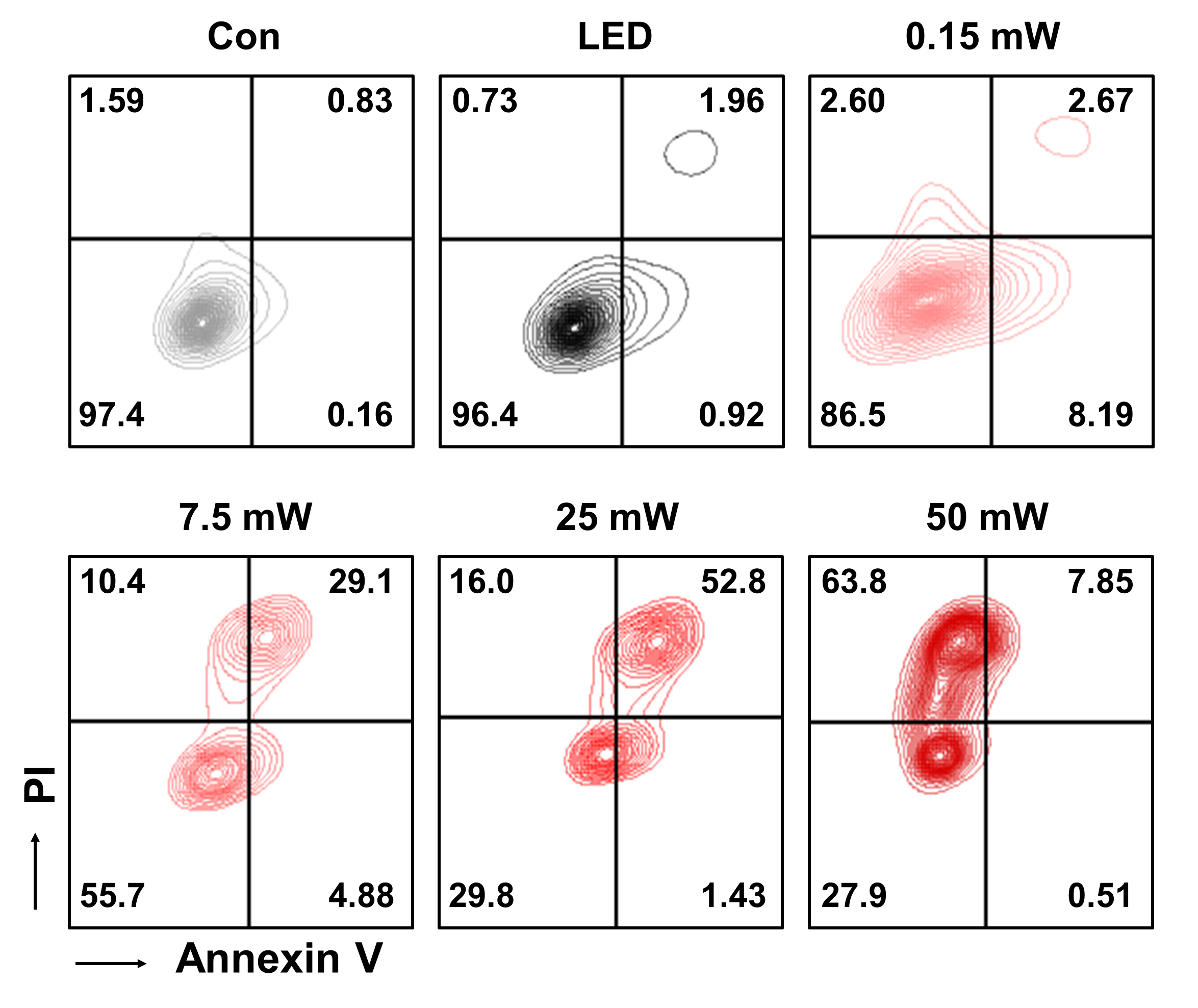


**Figure S5.** Annexin V/PI analysis of CT26 cells irradiated with different light intensities by micro-LED after 3 h of VPF treatment.


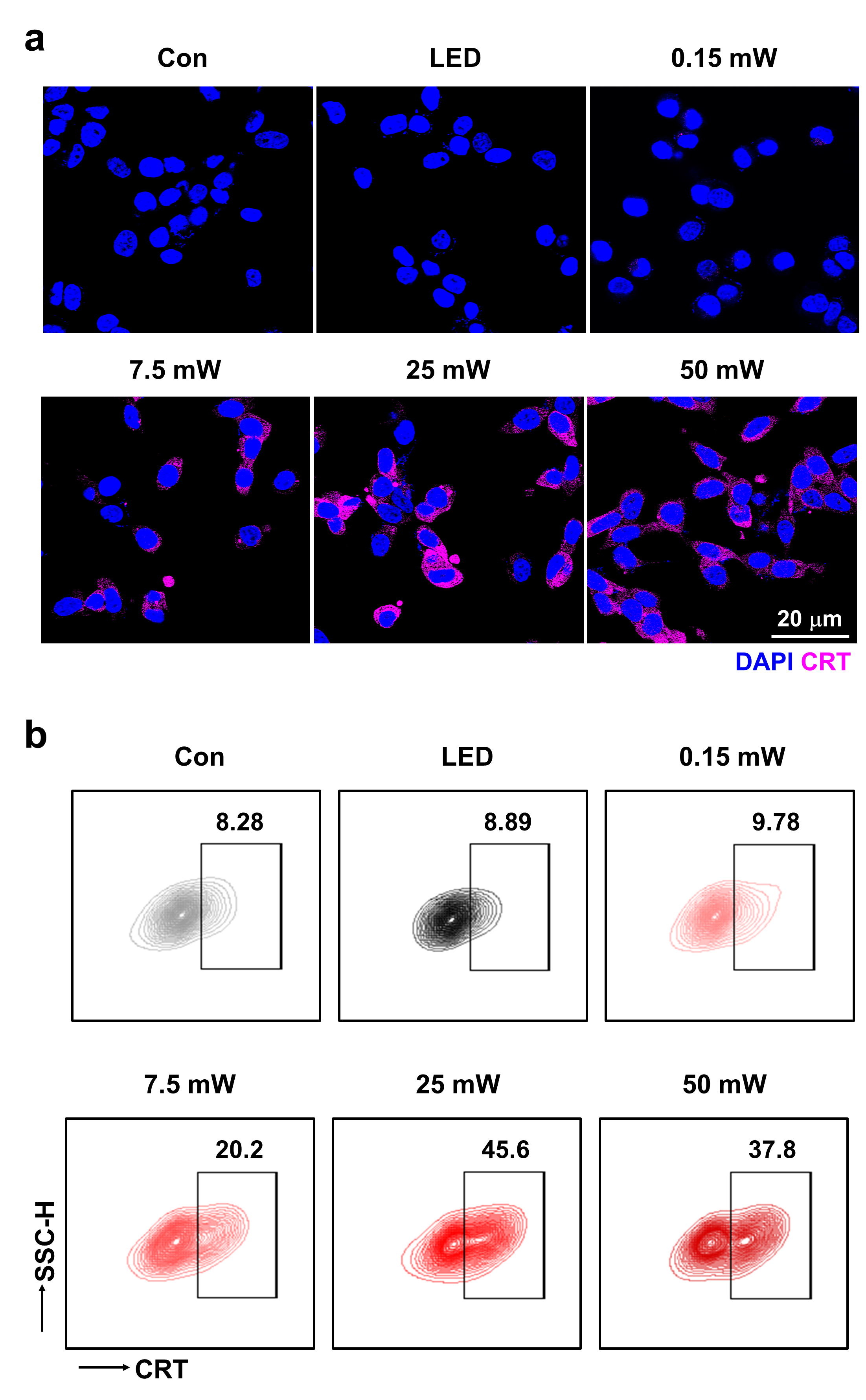


**Figure S6.** CRT expression of the CT26 cells irradiated with different light intensities by micro-LED after 3 h of VPF treatment, confirmed by fluorescence imaging and flow cytometry.


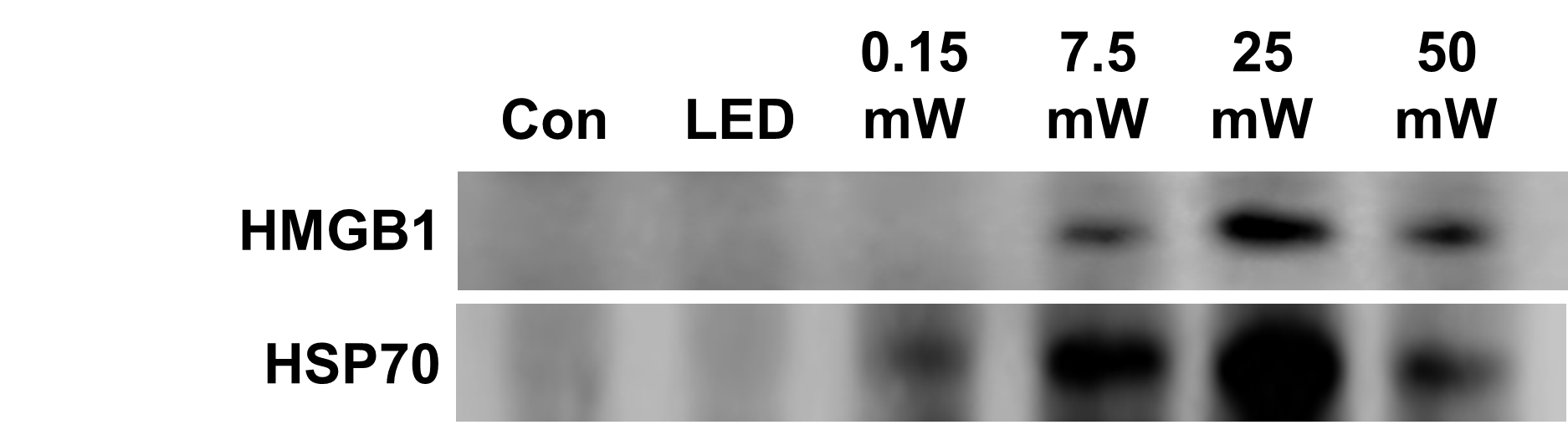


**Figure S7.** Extracellular release of HMGB1 and HSP70 from CT26 cells irradiated with different light intensities by micro-LED after 3 h of VPF treatment.

**
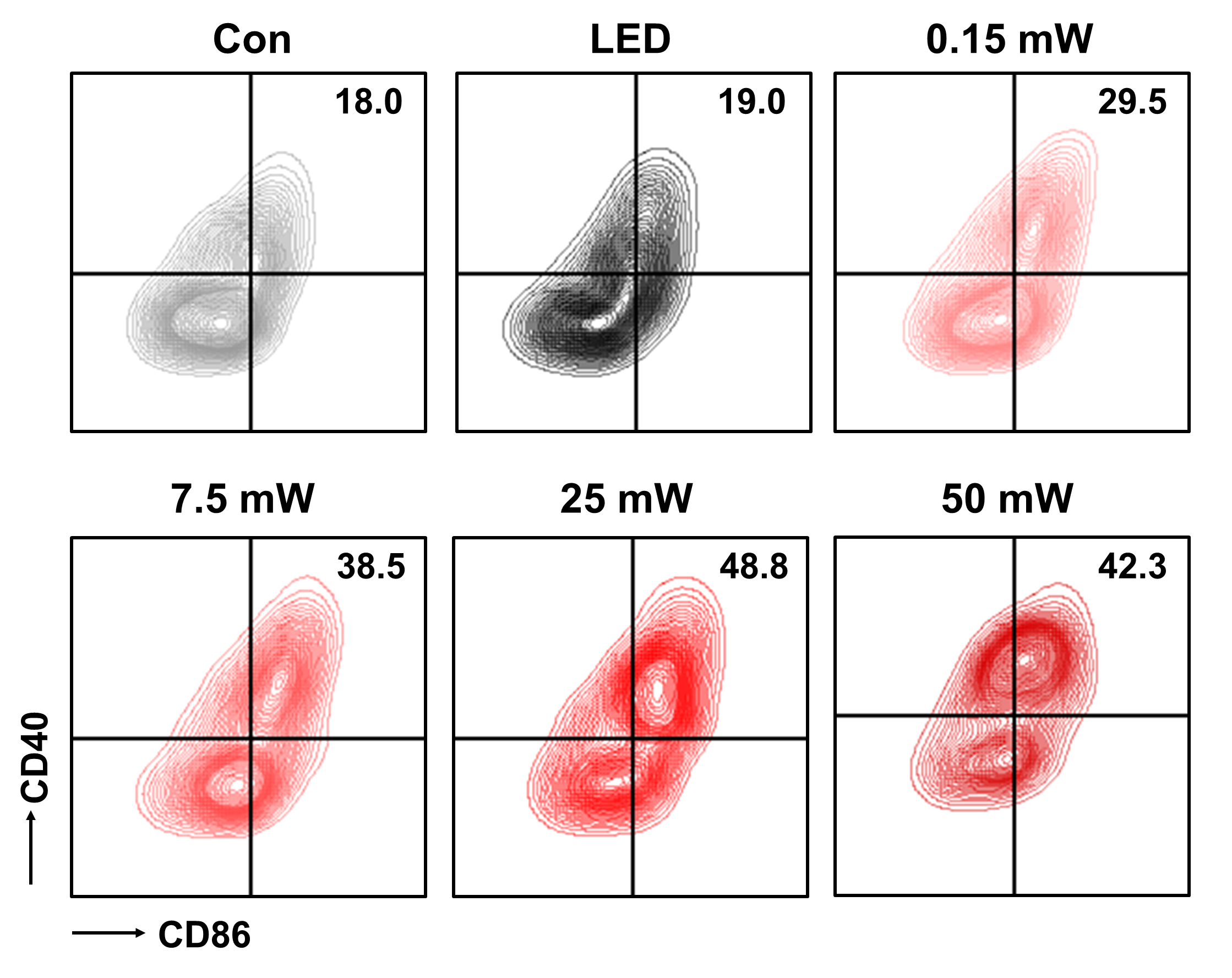
**

**Figure S8.** The percentage of mature DCs (CD11c^+^CD40^+^CD86^+^) in BMDCs after co-culture with culture medium containing CT26 cells irradiated with different light intensities by micro-LED after 3 h of VPF treatment.


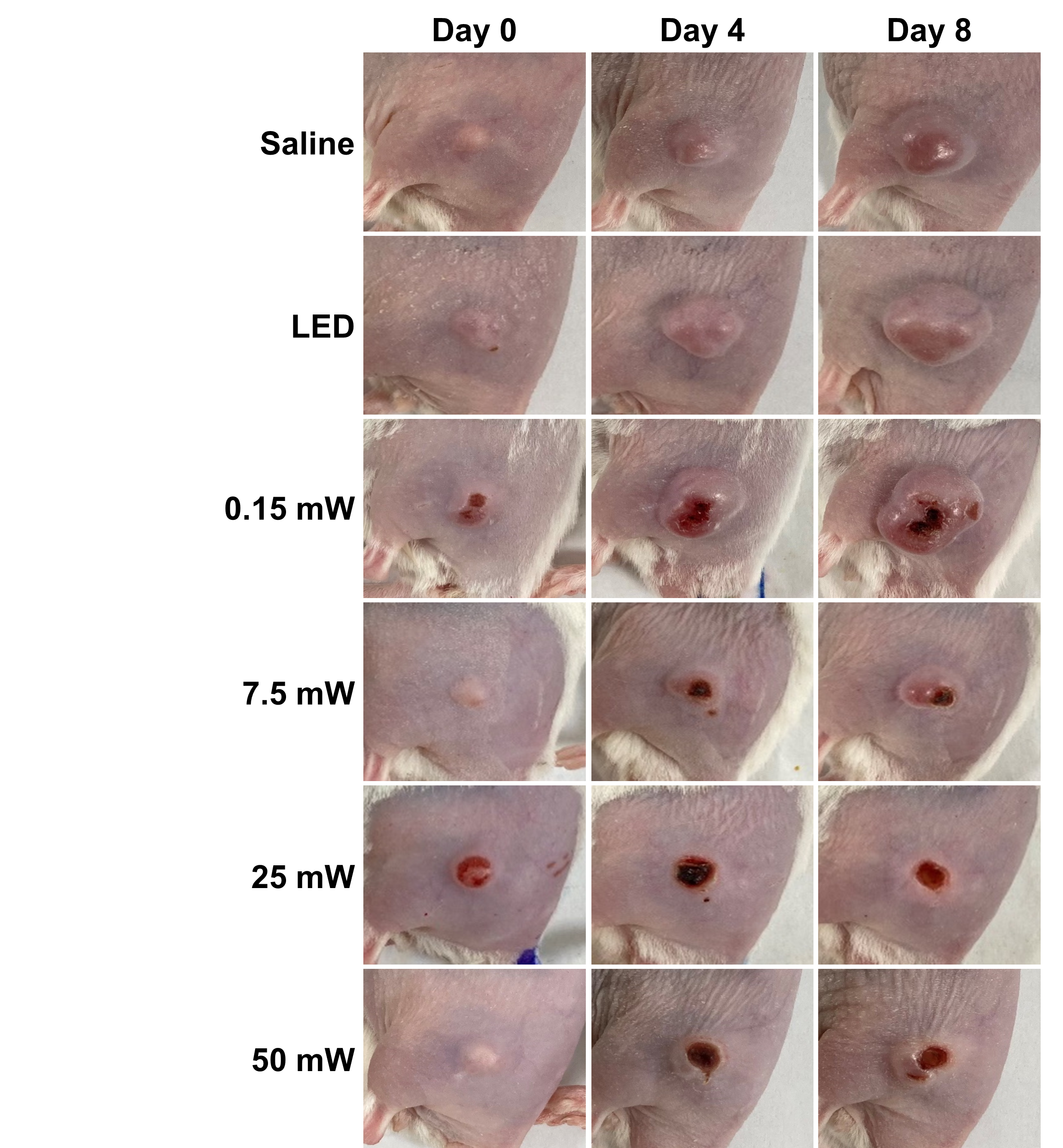


**Figure S9.** Optical images showing tumor volumes during micro-LED guided PDT at different light intensities.


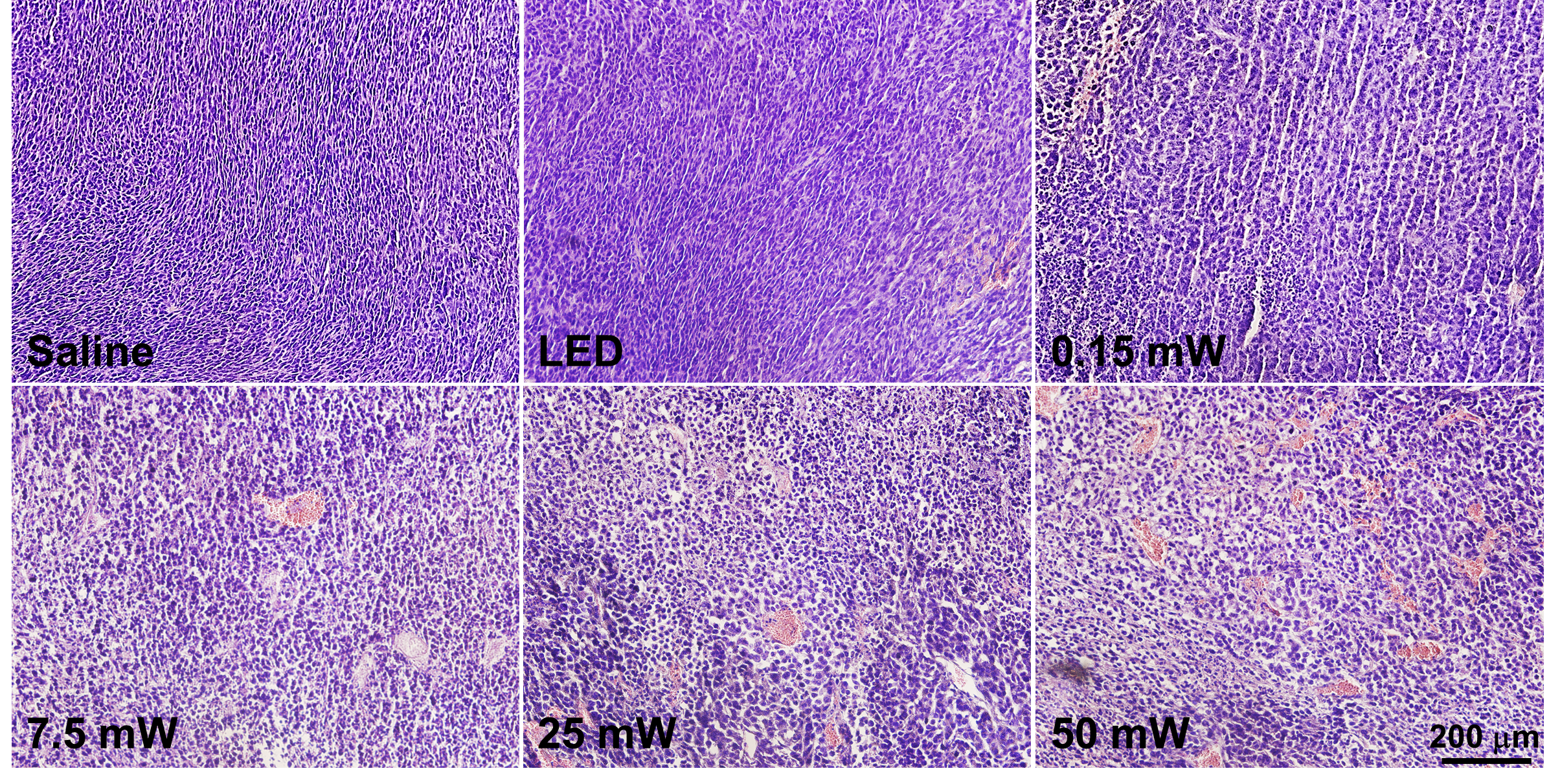


**Figure S10.** Tumor tissues stained with H&E on day 14 after treatment.


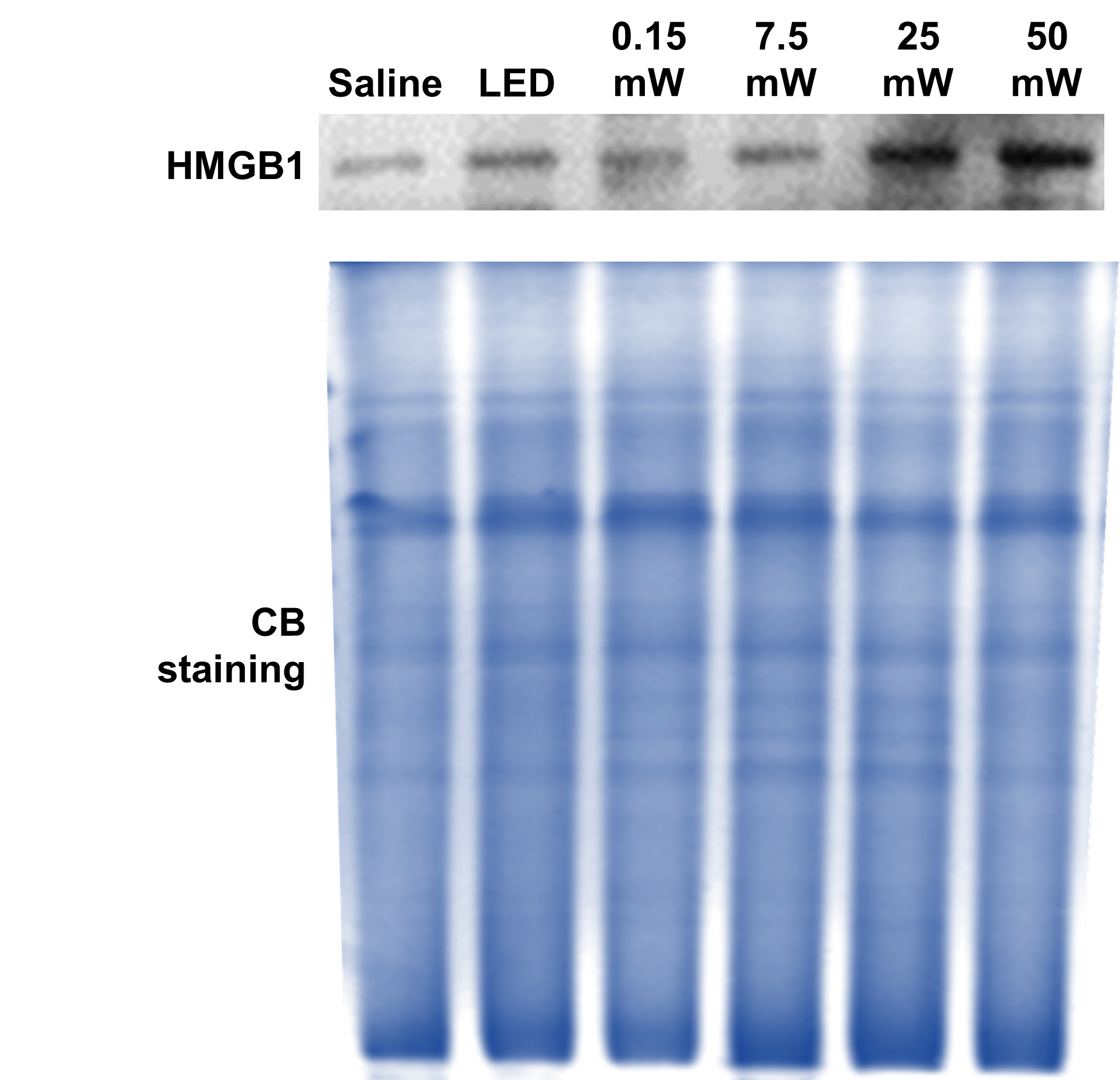


**Figure S11.** HMGB1 release from tumor cells to supernatants on day 14 after treatment.

**
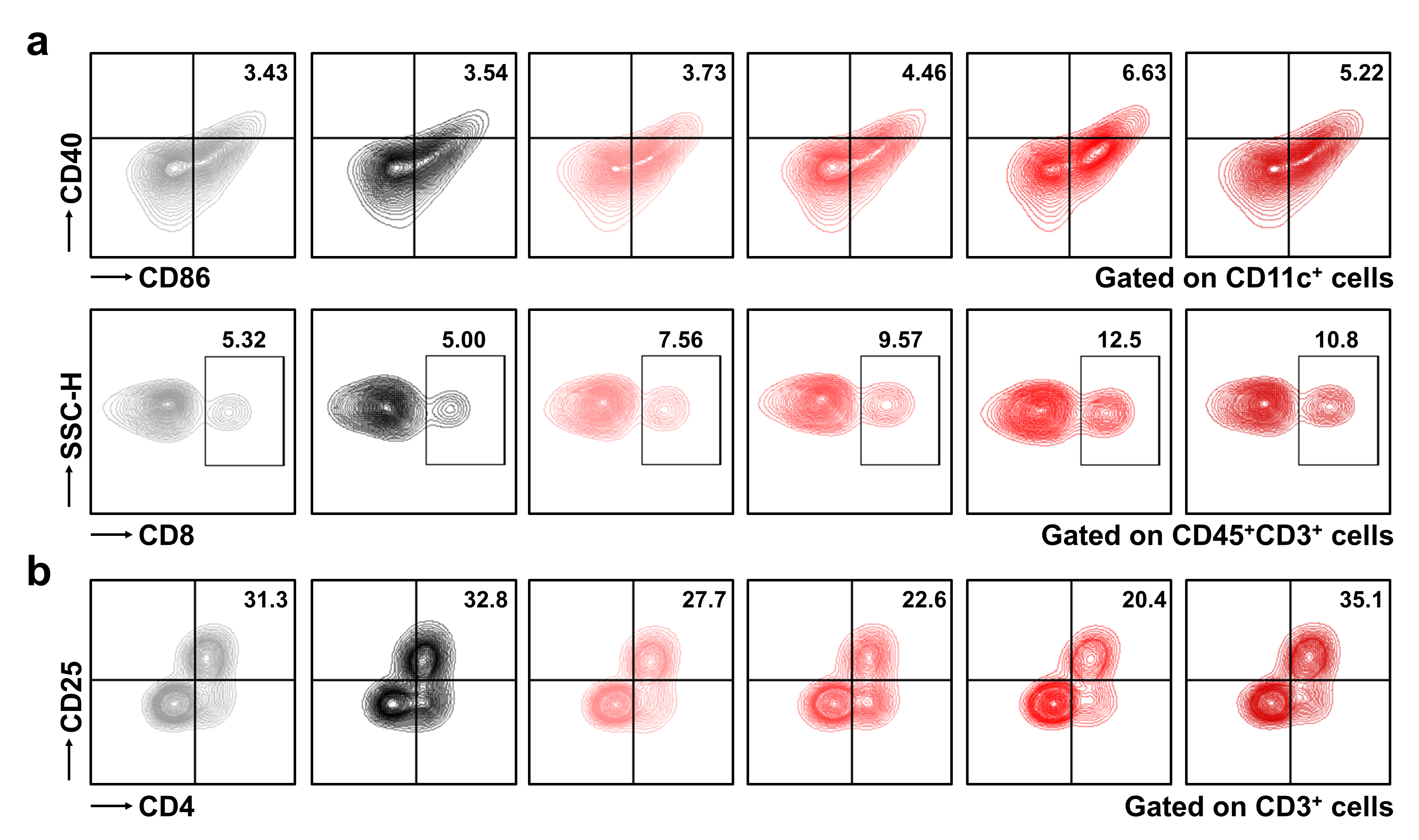
**

**Figure S12.** (a) Population of mature DCs (CD11c^+^CD40^+^CD86^+^) and cytotoxic T lymphocytes (CTLs; CD45^+^CD3^+^CD8^+^) in the tumor tissues on day 14 after treatment. (b) Population of regulatory T lymphocytes (Tregs; CD3^+^CD4^+^CD25^+^) in the tumor tissues on day 14 after treatment.

**
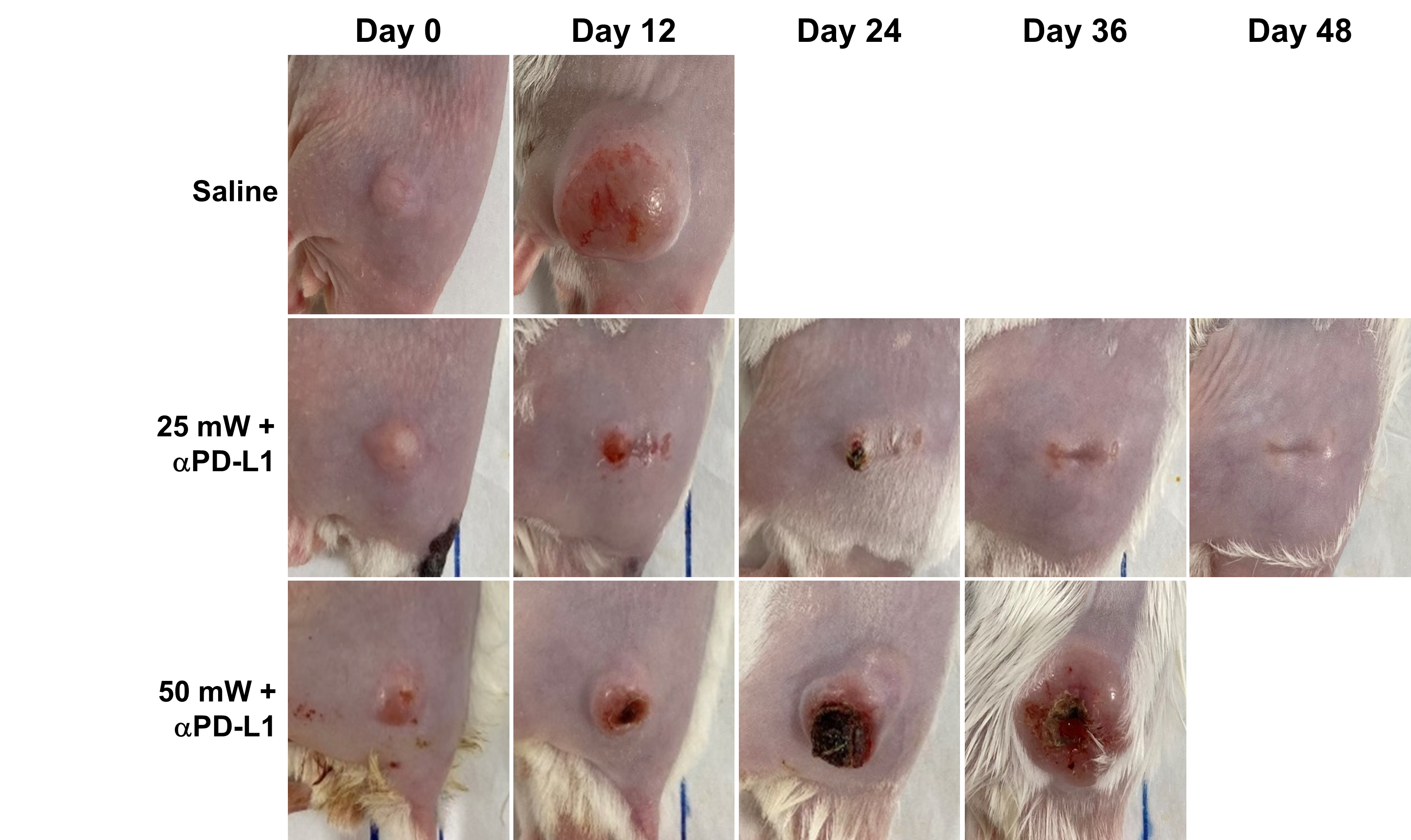
**

**Figure S13.** Optical images showing tumor volumes during co-treatment with micro-LED guided PDT (25 mW or 50 mW) and immune checkpoint blockade (anti-PD-L1 antibody; αPD-L1).

**
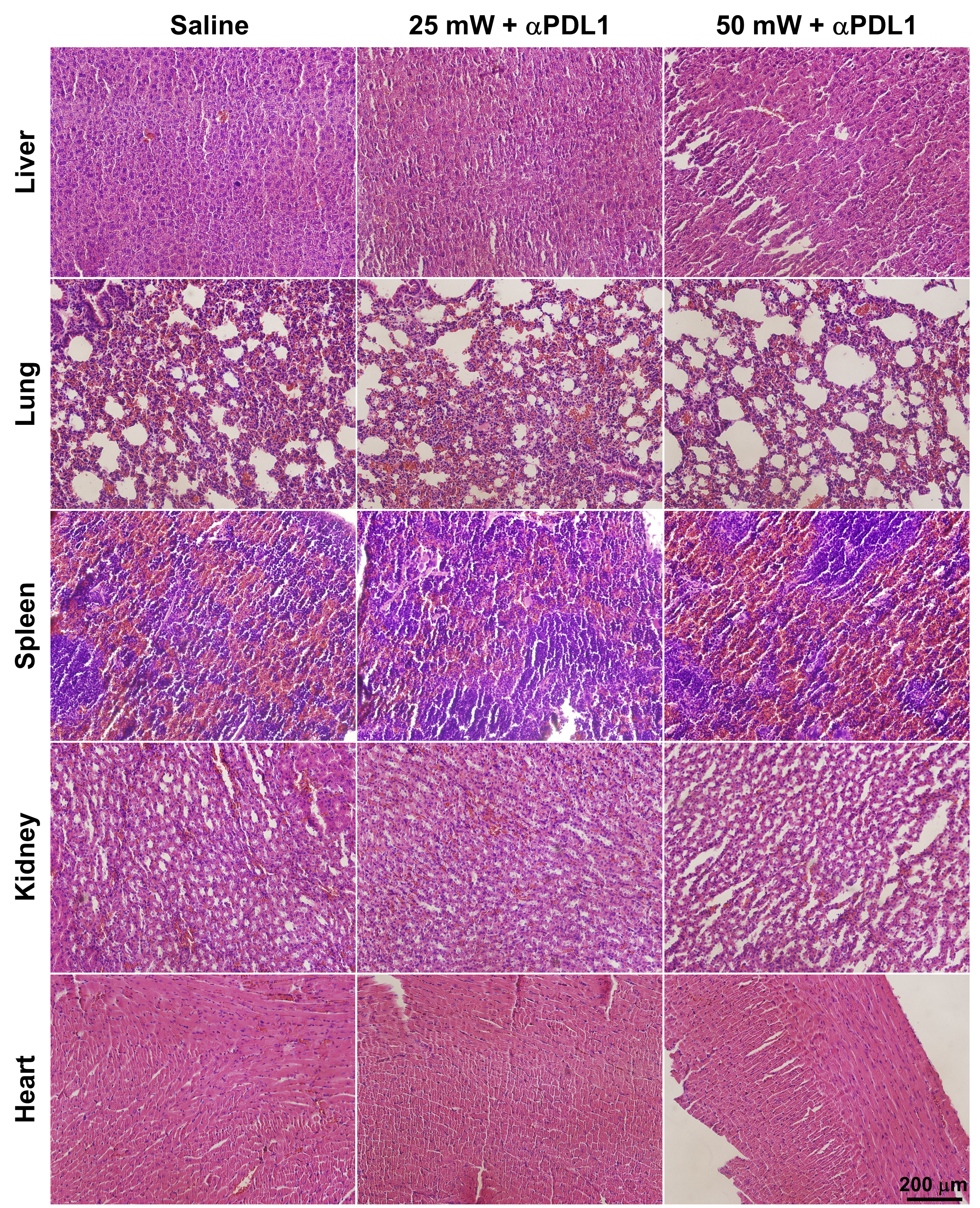
**

**Figure S14.** Toxicity study after co-treatment with micro-LED guided PDT (25 mW or 50 mW) and immune checkpoint blockade (anti-PD-L1 antibody; αPD-L1). The major organs were stained with H&E on day 14 after treatment.
