# Supplementary material for: Implantable micro-scale LED device guided photodynamic therapy to potentiate antitumor immunity with mild visible light
Source: Biomater Res. 2022 Oct 18;26:56. doi: 10.1186/s40824-022-00305-2 (PMC9580183; doi:10.1186/s40824-022-00305-2)
Supplement: Supplementary file 2 — Supplementary Material 2 [file 40824_2022_305_MOESM2_ESM.docx]

**Ewha Womans University**

Prof. Kwangmeyung Kim, Ph.D.

Chair Professor

Phone: +82-2-3277-2466

E-mail: kimkm@ewha.ac.kr

19 September, 2022

Dear Editor of Biomaterials Research,

At last 18 August 2022, we submitted our article entitled, **“Implantable micro-scale LED device guided photodynamic therapy to potentiate antitumor immunity with mild visible light”**, co-authored by, Jiwoong Choi, Il Seong Lee, Ju Seung Lee, Sangmin Jeon, Wan Su Yun, Suah Yang, Yujeong Moon, Jinseong Kim, Jeongrae Kim, Seunghwan Choy, Chanho Jeong, Man Kyu Shim, Tae-il Kim and Kwangmeyung Kim (Manuscript number: BMRS-D-22-00182).

On 16 September, 2022, we received the Editor’s letter for minor revision. The authors appreciated valuable comments. We did our best to perform additional experiments for finding the right answer for Reviewer’s comments. Now, we submit our revised manuscript containing additional results. The reviewer comments were carefully studied and reflected in the revised manuscript. Our responses to the reviewer comments are detailed summarized in bellow response letter.

We sincerely hope that you find this manuscript interesting and suitable for publication in your esteemed journal, **Biomaterials Research**. All the authors concur with the submission. The manuscript submitted for publication have not been previously reported and are not under consideration for publication elsewhere.

Sincerely,

------------------------------------------------------------------------

**Ewha Womans University**

College of Pharmacy, Graduate School of Pharmaceutical Sciences, Seoul 03760, Republic of Korea.

Kwangmeyung Kim, Ph.D.

Chair Professor

E-mail: [kimkm@ewha.ac.kr](mailto:kimkm@ewha.ac.kr)

**Responses to Reviewers’ Comments**

**Manuscript number: BMRS-D-22-00182 (*Biomaterials Research*)**

**Title:** Implantable micro-scale LED device guided photodynamic therapy to potentiate antitumor immunity with mild visible light

The authors appreciated all reviewers’ valuable comments. After a careful examination of those comments, we have prepared the revised manuscript to reflect them. Our responses to the reviewer’s comments are summarized as follows.

**Reviewer #1**: This paper has high originality and is recommended for publication after discussion with following two questions.

1. It has been suggested that the LED intensity can be adjusted by running the power of 3 LEDs. In such an environment, have you considered the effect of heat generated by the LED itself?

**Answer:** As the reviewer commented, assessing heat generation by the micro-LEDs is very important because a significant heat generation can negatively influence on inducing a potent antitumor immune response by resulting inflammatory responses due to necrotic cell death in the tumor tissues. To minimize the heat generation by devices, we designed the micro-LEDs with parallel array of four LEDs instead of a single large size one. Connecting several micro-LEDs in parallel can effectively control the heat generated without current drooping. Thus, the heat generated by micro-LED stacking four LEDs under operation power of 50 mW was only 6 ^o^C **(Added as Figure S2a in the revised manuscript)**. In addition, we already reported that the temperature change in the tumor tissues when the micro-LED devices were inserted at a depth of 3 mm from the surface of a thigh tumor and operated with 50 mW input power; in this experimental condition, the temperature in the tumor tissues rose to 34 ^o^C, which is much lower than the maximum temperature of general heating-based tumor therapies that can be safely used **(Figure 1g)**. In revised manuscript, we further directly evaluated the tissue damages by heat generated from micro-LED stacking four LEDs. When the tumor tissues were exposed to 50 mW input power of micro-LEDs, the tumor growth was not significantly different compared to that of non-treated mice, and tumor tissues stained with H&E or TUNEL also showed no significant structural abnormalities and apoptotic region **(Added as Figure S2b-d in the revised manuscript)**. These additional results and discussion were added in the revised manuscript.

**Text after correction:**

**1) (Introduction)** The implantable photonic devices stacking a number of micro-scale LED, which allow direct implantation to the target site by needle-like structure, are prepared to deliver therapeutic doses of light in the tumor tissues inaccessible by direct illumination of outer light source. Based on its needle-like structure, these devices can be directly implanted in the inner core part of the tumor tissues, thereby allowing light activation of photosensitizers in the whole tumor tissues. **In addition, the device is designed as stacking a number of LEDs with parallel array instead of a single large size one to minimize the heat generation during micro-LED guided PDT^16,17^.**

**2) (Figure 1) Next, the heat generation by micro-LED devices was assessed because a high temperature can negatively influence on inducing a potent antitumor immune response by resulting inflammatory responses due to necrotic cell death in the tumor tissues^9,10^. First, we assessed the heat generation by micro-LEDs under operation power of 50 mW, wherein the only 6 ^o^C was increased by the device (Figure S2a).** In addition, the temperature change was measured when the micro-LED devices were inserted at a depth of 3 mm from the surface of a thigh tumor and operated with 50 mW input power **(Figure 1g)**. Even with applied direct current to the four parallel LEDs, the temperature in the tumor tissues rose to 34°C. Since this is much lower than the maximum temperature of general heating-based tumor therapies that can be safely used, it can be expected that there is no damage to surrounding tissues due to the heat of the micro-LEDs. **As a result, when the tumor tissues were exposed to 50 mW input power of micro-LEDs, the tumor growth was not significantly different compared to non-treated mice for 15 days (Figure S2b). In addition, tumor tissues stained with H&E or TUNEL on day 15 after treatment also showed no significant structural abnormalities and apoptotic region (Figure S2c and S2d). These results clearly demonstrate that there are no significant damages in the tumor tissues by heat generation of micro-LEDs.**

**Figure S2 after correction:**

**Figure S2.** **(a)** Heat generation by micro-LEDs under input power of 50 mW. **(b)** The tumor growth of CT26 tumor-bearing mice when the tumor tissues were exposed to 50 mW input power of micro-LEDs. **(c-d)** The tumor tissues stained with **(c)** H&E or **(d)** TUNEL on day 15 after treatment.

**References after correction:**

[16] Kim, T.-i., Hyun Lee, S., Li, Y., Shi, Y., Shin, G., Dan Lee, S., Huang, Y., Rogers, J. A. & Su Yu, J. Temperature- and size-dependent characteristics in ultrathin inorganic light-emitting diodes assembled by transfer printing. *Applied Physics Letters* **104**, 051901 (2014).

[17] Kim, T.-i., Jung, Y. H., Song, J., Kim, D., Li, Y., Kim, H.-s., Song, I.-S., Wierer, J. J., Pao, H. A., Huang, Y. & Rogers, J. A. High-Efficiency, Microscale GaN Light-Emitting Diodes and Their Thermal Properties on Unusual Substrates. *Small* **8**, 1643-1649 (2012).

**2**. It would be nice if you could distinguish whether the LED device was experimentally evaluated by implanting the LED device into the tumor tissue or by irradiation onto the skin.

**Answer:** In present study, we designed the photonic devices as a needle-like structure, which can be implanted in the inner core part of the tumor tissues for delivering therapeutic doses of the light to the deep inside the tumors. This is to overcome the depth limitation of visible light that cannot penetrate the surface of the biological tissues by more than 5-10 mm for inducing a potent antitumor immune response by improving the efficacy of PDT. In the revised manuscript, we added more detailed discussion about the strategy of micro-LEDs-mediated PDT for clear understanding of the readers.

**Text after correction:**

**(Introduction)** The implantable photonic devices stacking a number of micro-scale LED, which allow direct implantation to the target site by needle-like structure, are prepared to deliver therapeutic doses of light in the tumor tissues inaccessible by direct illumination of outer light source. **Based on its needle-like structure, these devices can be directly implanted in the inner core part of the tumor tissues, thereby allowing light activation of photosensitizers in the whole tumor tissues.**

**Reviewer #2**: This study deals with the photodynamic therapy with implantable micro-scale LED device. The authors focused on finding proper intensity of micro-LEDs through confirming the rate of early apoptosis, late apoptosis, and necrosis of CT26 cells. In addition, authors applied immune checkpoint blockade with micro-LEDs, which showed 100% tumor regression in 25 mW-treated group. This research can be useful for approaching effective cancer therapy.

**1**. Please explain the mechanism of VPF on cancer cells in Introduction of manuscript.

**Answer:** As the reviewer commented, the detail mechanism of photodynamic therapy by VPF was added in the Introduction part of the revised manuscript.

**Text after correction**

**(Introduction)** Following the intravenous administration of a photosensitizer (verteporfin; VPF), the optimal intensity and period of light exposure by micro-LED implanted in the tumor tissues are investigated to promote antitumor immunity by inducing potent ICD in tumor cells **(Scheme 1b)**. **The VPF has long been clinically used for PDT, which causes photochemical damage to the mitochondria by promoting reactive oxygen species (ROS) upon visible light irradiation with wavelength of a 690 nm^18^.** **Scheme 1c** shows the different cancer cell death mechanism using micro-LED guided PDT depending on optimal, less or over light intensity.

**Reference after correction**

[18] Lim, T. H., Lai, T. Y. Y., Takahashi, K., Wong, T. Y., Chen, L.-J., Ruamviboonsuk, P., Tan, C. S., Lee, W. K., Cheung, C. M. G., Ngah, N. F., Patalauskaite, R., Margaron, P., Koh, A. & for the, E. I. I. S. G. Comparison of Ranibizumab With or Without Verteporfin Photodynamic Therapy for Polypoidal Choroidal Vasculopathy: The EVEREST II Randomized Clinical Trial. *JAMA Ophthalmology* **138**, 935-942 (2020).

2. Page 2 of 29) Please correct the verb 'causes' to cause.

**Answer:** As the reviewer commented, the authors double-checked and corrected the typo errors throughout the manuscript.

**Text after correction:**

**(Figure 4)** This is attributable to severe inflammatory responses due to necrotic cell death in the tumor tissues, which **cause** the activation of Tregs, due to over light intensity.

**3**. Authors should check the caption of Figure 2(c). Figure 2(c) means the viability of the CT26 cells with different light intensities from micro-LED, not in terms of a number of LEDs.

**Answer:** As the reviewer commented, the caption of the Figure 2c was corrected in the revised manuscript.

**Text after correction:**

**(Figure 2c caption)** The viability of the CT26 cells irradiated **with different light intensities by micro-LED after 3 h of VPF treatment.**

**4**. Authors should add precisely the type of staining with S13.

**Answer:** As the reviewer commented, an information for the staining type of the histological analyses in the Figure S13 was added in the revised manuscript.

**Text after correction:**

**1) (Figure 5)** During treatments, significant body weight changes and toxicity in the major organs were not observed in all groups, **wherein the structural abnormalities in the major organs were assessed after H&E staining** **(Figure 5c and S14)**.

**2) (Figure S14 caption)** Toxicity study after co-treatment with micro-LED guided PDT (25 mW or 50 mW) and immune checkpoint blockade (anti-PD-L1 antibody; αPD-L1). **The major organs were stained with H&E on day 14 after treatment.**
